# A Glucose‐Responsive Intelligent Antibacterial and Oxygen‐Producing Hydrogel Promotes the Healing of Diabetic Wounds by Regulating Cellular Heterogeneity

**DOI:** 10.1002/advs.202517028

**Published:** 2026-01-31

**Authors:** Manxuan Liu, Zhongcheng Li, Qian Ren, Ziqian Lu, Yubing Zhang, Yili Guo, Ruomeng Li, Die Hu, Linglin Zhang

**Affiliations:** ^1^ State Key Laboratory of Oral Diseases & National Center for Stomatology & National Clinical Research Center for Oral Diseases West China Hospital of Stomatology Sichuan University Chengdu P. R. China; ^2^ Department of Endodontics West China Hospital of Stomatology Sichuan University Chengdu P. R. China

**Keywords:** diabetic infected wound repair, metal phenolic network, oxygen‐producing hydrogel, glucose‐responsive, single‐cell RNA sequencing

## Abstract

The chronic and refractory infected wounds of diabetes are primarily attributed to the persistent bacterial infection and the inhibition of wound healing caused by hypoxia. Hydrogel with intelligent drug delivery systems hold significant potential in the treatment of diabetic wounds. Herein, we have developed an glucose‐responsive intelligent hydrogel named as CF‐CPGaMPN, which incorporates polyvinylpyrrolidone‐coated calcium peroxide (PVP@CaO_2_) nanoparticles, catalase, and gallium‐polyphenol (GaMPN) nanoparticles. The borate ester bonds in the CF‐CPGaMPN hydrogel break under high glucose conditions, releasing GaMPN nanoparticles, thereby achieving glucose‐triggered on‐demand drug release. The CF‐CPGaMPN hydrogel not only inhibits various microorganisms but also continuously releases oxygen, thereby promoting the healing of diabetic infection wounds. Furthermore, the multicellular ecosystem surrounding the CF‐CPGaMPN hydrogel is also explored, and the diverse cellular heterogeneity is analyzed by single‐cell RNA sequencing, highlighting the critical roles of Neutrophils, Fibroblasts, and Epidermal cells in diabetic infected wound. In addition, CF‐CPGaMPN hydrogel inhibits the Neutrophil extracellular trap (NET) formation and alleviates the cellular hypoxic environment to improve diabetic wound healing. In conclusion, the CF‐CPGaMPN hydrogel not only provides a promising drug release strategy for the healing of diabetic infected wounds, but also contributes to the rational design of customized hydrogels for biomedical use targeting different cellular functions.

## Graphical abstract

We have developed a glucose‐triggered on‐demand drug delivery CF‐CPGaMPN hydrogel based on borate ester bonds. It inactivates microbes, releases oxygen, and enables on‐demand drug release in high‐glucose environments to promote healing of diabetic wounds. Single‐cell sequencing reveals that the CF‐CPGaMPN hydrogel significantly alleviates dysfunctional neutrophils, fibroblasts, and epidermal cells in the diabetic microenvironment, elucidating the key molecular mechanisms of different cell subpopulations in diabetic wound healing.

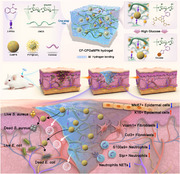

## 1 Introduction

Diabetes mellitus is a chronic metabolic disorder affecting 536.6 million people globally, with a predicted increase in prevalence to 783.2 million by 2045 [1]. Approximately 25% of diabetic patients experience delayed and non‐healing wounds, leading to prolonged pain and even lower extremity amputations [2, 3]. This clinical problem mainly stems from two interrelated core pathological factors: bacterial infection and tissue hypoxia [4]. Therefore, there is still an urgent need for new therapies aimed at combating bacterial infections in wounds and alleviating hypoxia in wounds to accelerate the healing of diabetic infected wounds.

Infection stands as the primary reason for delayed wound healing in diabetes. Sustained hyperglycemia creates an environment that supports bacterial proliferation and invasion, leading to chronic and non‐healing wounds. Therefore, controlling bacterial infection is essential for the effective healing of diabetic wounds [5]. In combating bacterial infections in diabetic wounds, antibiotic therapy remains the clinical gold standard for infection management [6]. Nevertheless, the escalating problem of antibiotic misuse has fueled a growing phenomenon of multidrug‐resistant bacterial strains. It is estimated that by 2050, the number of annual deaths caused by multi‐drug resistant bacteria will reach 10 million [7]. Consequently, numerous studies have focused on developing novel antimicrobial agents that are distinct from traditional antibiotics, including nanoenzymes [8, 9], metal or metal oxide nanoparticles [10, 11], metal‐organic frameworks [12, 13], cationic polymers [14, 15], dendrimeric peptides [16, 17], carbon‐based nanostructures [18], nanocellulose‐based materials [19], and supramolecular complexes [20, 21]. Metal‐phenolic networks (MPN), which are based on the coordination between polyphenols and metal ions, have attracted considerable interest in the field of functional biomaterial design [22]. MPN offer several advantages such as simple fabrication, abundant sources, and a bioactive combination of polyphenols and metal ions [23, 24]. Among the metal ions used to form MPN, Ga^3+^ is notable for its remarkable antibacterial activity. Due to the similar properties of gallium and iron on ionic radius, ionization potential, and electron affinity, bacterial uptake mechanisms cannot distinguish between gallium and iron [25]. Gallium cannot be reduced in physiological environments, whereas iron‐related biological activities rely on the redox cycling of iron. Consequently, gallium disrupts iron‐dependent biological processes [26]. By substituting iron with gallium, bacterial iron metabolism is impaired, leading to decreased bacterial survival rates in vitro [27, 28]. Additionally, bacteria find it difficult to evolve resistance to gallium [29]. However, the antibacterial effect of inhaled free Ga^3+^ is limited due to rapid clearance by macrophages [30]. The formation of GaMPN nanoparticles with tannic acid effectively reduces macrophage clearance. Moreover, bacterial siderophores, which are responsible for iron transport, possess a polyphenolic structure [31]. When Ga^3+^ acts synergistically with tannic acid, the bacterial absorption of Ga^3+^ is enhanced, thereby augmenting its antibacterial properties [32]. However, the rapid chelation process of MPN nanoparticles poses a challenge [33]. To prevent the rapid chelation of MPN nanoparticles, coordination regulators such as Diethyldithiocarbamate (DEDTC) are often used as chelating agents for MPN due to their strong metal chelating ability, low cytotoxicity, and antibacterial properties [34, 35].

Hypoxia is another important factor that hinders the healing of diabetic wounds. Due to the high glucose environment, metabolic demands, and poor blood circulation, wounds often suffer from insufficient oxygen supply [36]. When an injury occurs, vascular defects and the accumulation of high oxygen‐consuming cells further exacerbate tissue hypoxia at the wound site [37]. Hypoxia impairs wound healing through the suppression of angiogenesis, re‐epithelialization, and Extracellular Matrix (ECM) synthesis [38]. Existing therapeutic approaches to enhance oxygen supply in chronic diabetic wounds include hyperbaric oxygen treatment, methods that promote capillary formation(such as stem cell delivery for angiogenic factor expression) and the application of oxygen‐carrying biomaterials mimicking hemoglobin [39, 40, 41]. However, these methods are constrained by inadequate oxygen penetration and relatively rapid oxygen release [42, 43]. Therefore, new treatment strategies need to be sought in order to significantly improve the hypoxic microenvironment within diabetic wounds. As a metal oxide, calcium peroxide (CaO_2_) can react with H_2_O under the condition of catalase, and the generated oxygen improves tissue hypoxia [44]. By introducing polyvinylpyrrolidone (PVP) as a stabilizer to coat CaO_2_ nanoparticles, nanoparticles with uniform and controllable sizes can be prepared [45]. Subsequently, these nanoparticles were combined with highly biocompatible hydrogels to construct a composite system capable of continuously releasing oxygen. This strategy not only significantly prolonged the oxygen release time [46], but also research has confirmed that the oxygen released from the oxygen carriers in the hydrogel has a much deeper tissue penetration depth than that of directly using gas oxygen [47], thereby more effectively alleviating tissue hypoxia and promoting repair.

In addition, traditional hydrogel dressings are characterized by uncontrollable drug release, which increases treatment costs and reduces the efficacy of the hydrogel dressing [48]. Stimuli‐responsive hydrogels have garnered considerable interest in recent years due to their ability to react to external cues—including reactive oxygen species (ROS), temperature, pH, and enzymes—allowing for precise regulation of drug release [49]. The controlled delivery system that releases bioactive substances targeting the specific microenvironment of wounds can effectively promote wound healing [50]. Unlike normal wounds, diabetic wounds are characterized by elevated blood glucose levels [51]. Hence, the design of an intelligent hydrogel system that specifically responds to glucose concentration in the microenvironment, enabling on‐demand release of antibacterial drugs and hypoxia relief, is crucial for the healing of diabetic infected wounds.

**FIGURE 1:**
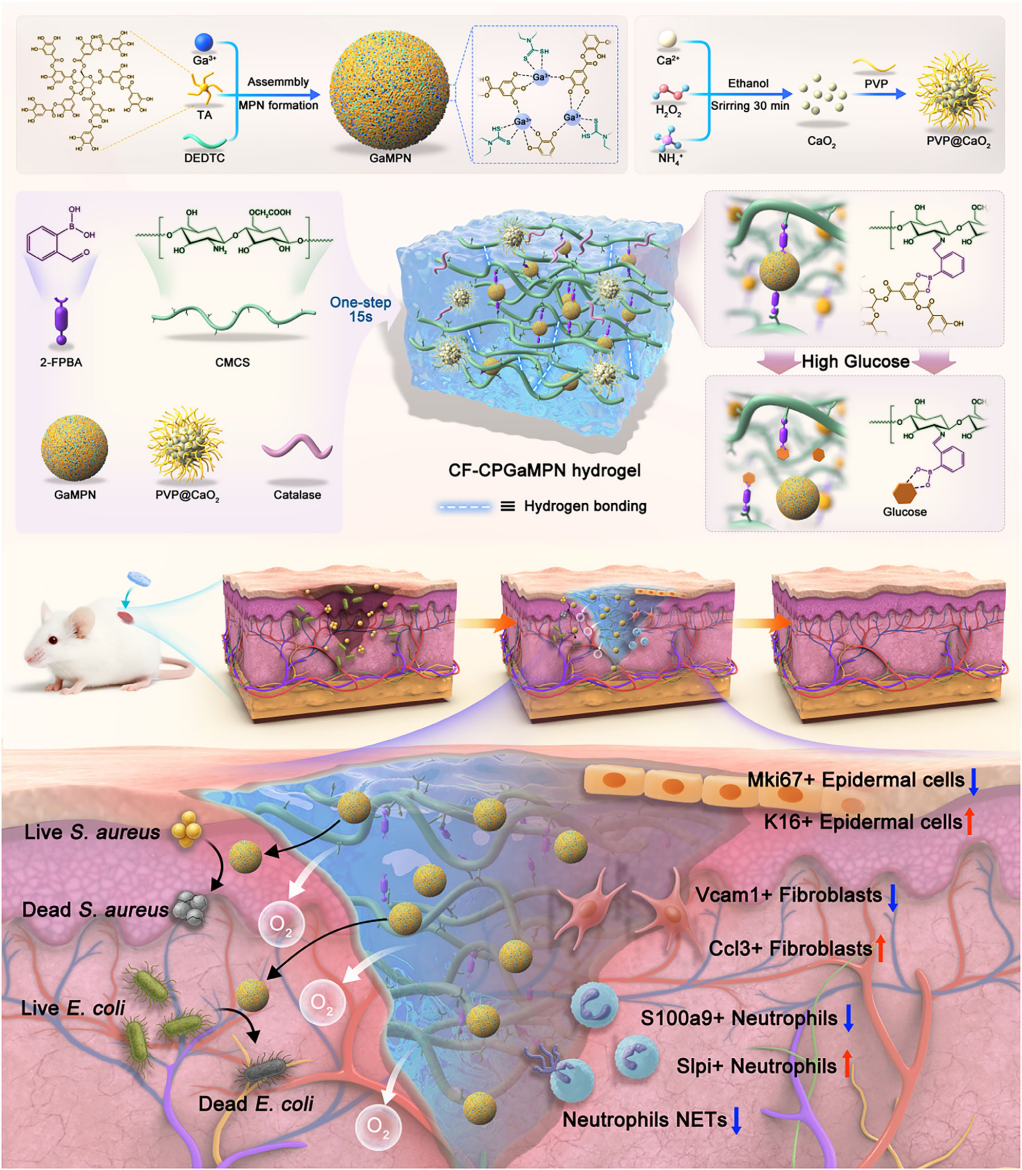
Schematic diagram of the synthesis of CF‐CPGaMPN hydrogel and its treatment of diabetic infected wounds.

Based on the aforementioned analysis, we developed an on‐demand drug release CF‐CPGaMPN hydrogel system dependent on blood glucose concentration, which solved the problems of bacterial infection and hypoxia in diabetic wounds to promote the healing of diabetic infected wounds, and innovatively explored its further mechanism of regulating cell behavior in diabetic wounds (Figure 1). The CF‐CPGaMPN hydrogel is formed by incorporating GaMPN nanoparticles, polyvinylpyrrolidone‐coated calcium peroxide (PVP@CaO_2_) nanoparticles, and catalase into a self‐healing hydrogel composed of carboxymethyl chitosan (CMCS) and 2‐formylphenylboronic acid (2‐FPBA). The self‐healing property of the hydrogel facilitates its adaptability to various irregular wound surfaces and allows for recovery from hydrogel damage. Glucose in the diabetic wound microenvironment serves as a “smart key” to activate drug release. The boronate ester bonds in the hydrogel system cleave in a high‐glucose environment, releasing the encapsulated GaMPN nanoparticles from the hydrogel and achieving glucose‐triggered on‐demand drug release. GaMPN nanoparticles exhibit antibacterial properties against both Gram‐positive and Gram‐negative bacteria, effectively disrupting the formation of bacterial biofilms and promoting the healing of diabetic infected wounds. Simultaneously, PVP@CaO_2_ nanoparticles and catalase are loaded into the hydrogel. Following implantation into diabetic wounds, CaO_2_ is gradually released from the hydrogel and undergoes hydrolysis in the tissue fluid, yielding calcium hydroxide and H_2_O_2_. The resulting H_2_O_2_ is subsequently decomposed by catalase into oxygen and water; this continuous oxygen release helps ameliorate local hypoxia in the wound tissue. The introduction of single‐cell RNA sequencing (scRNA‐seq) has further advanced diabetic wound healing research, offering new insights into cellular functions, pathological mechanisms, and the complex wound microenvironment. This technology can accurately delineate cellular subpopulations, elucidate key molecular mechanisms, and identify new therapeutic targets [52]. Overall, the application of scRNA‐seq in diabetic wounds offers new insights into the mechanisms by which dressings promote diabetic wound healing and potential directions for further treatment. Therefore, we studied the mechanism by which CF‐CPGaMPN hydrogel regulates the behavior of diabetic wound cells through scRNA‐seq technology. In summary, the CF‐CPGaMPN hydrogel proposed in this study offers a novel solution for the repair of infected diabetic wounds, significantly accelerating the wound healing process. By elucidating its wound‐healing mechanisms, this research provides novel insights and strategies for the customized design and evaluation of biomaterials that promote diabetic wound healing in future.

## 2 Results and Discussion

### 2.1 Preparation and Characterization of GaMPN Nanoparticles

**FIGURE 2:**
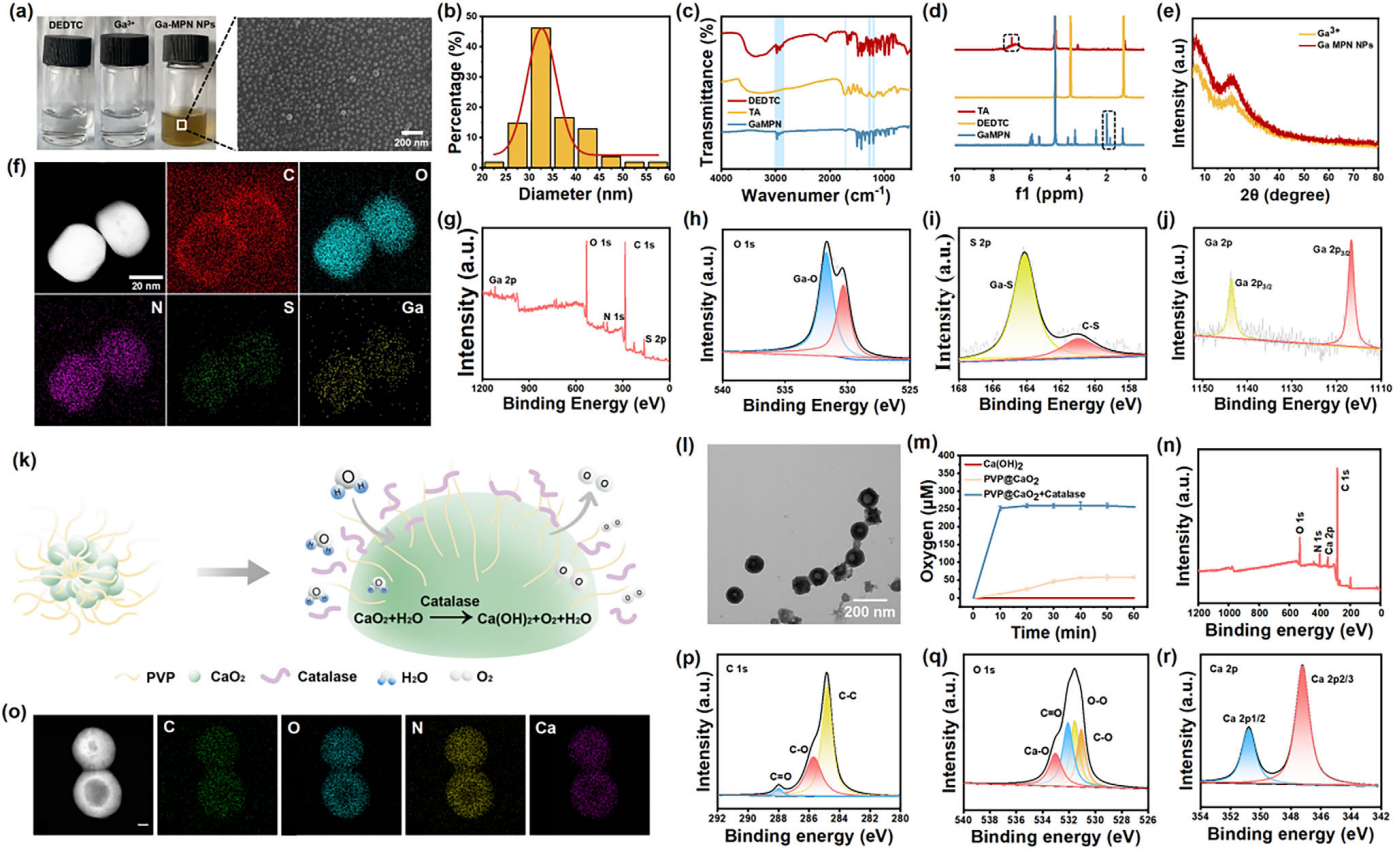
Characterization of GaMPN nanoparticles and PVP@CaO_2_ nanoparticles. (a) Photographs of solutions of DEDTC, Ga^3+^ and GaMPN nanoparticles. (b) Size distribution map of GaMPN nanoparticles. (c) FT‐IR spectra of TA, DEDTC and GaMPN nanoparticles. (d) ^1^H NMR spectra of TA, DEDTC and GaMPN nanoparticles. (e) XRD patterns of Ga^3+^ and GaMPN nanoparticles. (f) Element mapping of GaMPN nanoparticles. (g) XPS survey scan of GaMPN nanoparticles. (h) O 1s spectra of GaMPN nanoparticles. (i) S 2p spectra of GaMPN nanoparticles. (j) Ga 2p spectra of GaMPN nanoparticles. (k) Schematic representation of PVP@CaO_2_ nanoparticles and their oxygen release mechanism. (l) Transmission electron microscopy images of PVP@CaO_2_ nanoparticles. (m) Comparison of releasing oxygen between CaO_2_ and PVP@CaO_2_ nanoparticles with or without catalase. (n) XPS survey scan of PVP@CaO_2_ nanoparticles. (o) Element mapping of PVP@CaO_2_ nanoparticles. (p) C 1s spectra of PVP@CaO_2_ nanoparticles. (q) O 1s spectra of PVP@CaO_2_ nanoparticles. (r) Ca 2p spectra of PVP@CaO_2_ nanoparticles.

The molecular formulae for the synthesis of GaMPN nanoparticles are displayed in Figure S1a. Scanning Electron Microscopy (SEM) analysis showed that the GaMPN nanoparticles were well dispersed and spherical (Figure 2a). The surface morphology of GaMPN nanoparticles was further observed by atomic force microscopy (AFM) (Figure S1c), with the same results as those observed by SEM. The size of the GaMPN nanoparticles was quantified by particle size distribution plots, showing that these nanoparticles were uniform in size, with 77% of their diameters distributed in the 25∼40 nm interval (Figure 2b). The presence of C = O peak at 1713 cm^−1^, C‐N peak at 1270 cm^−1^, C‐O peak at 1200 cm^−1^, and ‐CH_3_ peak at 2970 cm^−1^ were observed on the Fourier Transform Infrared Spectroscopy (FT‐IR) spectra of GaMPN nanoparticles, which confirms the presence of DEDTC and TA in GaMPN nanoparticles (Figure 2c). In addition, the chemical shift peaks appearing at 2.0 and 1.8 ppm on the ^1^H NMR spectra of GaMPN nanoparticles indicate hydrogen atoms on SH, proving the successful grafting of DEDTC. Whereas, the chemical shift peak appearing at 6.9 ppm for TA on the ^1^H NMR spectrum of GaMPN nanoparticles was absent, which was attributed to the substitution of Ga for H on the o‐hydroxyl group of TA benzene ring, further proving the successful synthesis of GaMPN nanoparticles (Figure 2d). GaMPN nanoparticles showed the same X‐Ray Diffraction (XRD) patterns as free Ga^3+^, indicating the absence of oxidation of Ga^3+^ in GaMPN nanoparticles as well as the amorphous nature of GaMPN (Figure 2e). The elements mapping showed that C, N, O, S, and Ga were uniformly distributed in GaMPN nanoparticles (Figure 2f). The composition and coordination environment of the GaMPN nanoparticles were further characterized by X‐ray photoelectron spectroscopy (XPS). The measured spectra confirmed the presence of Ga, O, and S from three different compositions (i.e., Ga^3+^, TA, and DEDTC) (Figure 2g), with peaks at 531.7, 164.1, 1143.6, and 1116.7 eV belonging to Ga‐PhOH (signal from O 1s), Ga‐S (signal from S 2p), Ga‐S (signal from Ga 2p1/2) and Ga‐S (signal from Ga 2p3/2) ligand bonds, respectively (Figure 2h–j).

### 2.2 Preparation and Oxygen Release of PVP@CaO2 Nanoparticles

We used PVP to aggregate multiple CaO_2_ nanoparticles together to form PVP@CaO_2_ nanoparticles (Figure 2k). The molecular formulae for the synthesis of PVP@CaO_2_ nanoparticles are displayed in Figure S1b. First, the morphology of PVP@CaO_2_ nanoparticles was observed by SEM. As shown in Figure 2l, the PVP@CaO_2_ nanoparticles were uniform in size, and 65% of the nanoparticles had diameters of 60∼90 nm (Figure S1d). Then the elements in PVP@CaO_2_ nanoparticles were detected by EDS. As shown in Figure 2o, the elements C, N, O and Ca were uniformly distributed in PVP@CaO_2_ nanoparticles, confirming the successful synthesis of PVP@CaO_2_ nanoparticles. The FT‐IR spectrum of PVP@CaO_2_ nanoparticles showed an absorption peak near 1646 cm^−1^ of C = O peak, indicating the presence of PVP. The absorption peak at 875 cm^−1^ represents the O‐O peak indicating the presence of CaO_2_ nanoparticles (Figure S1e). X‐ray photoelectron spectroscopy (XPS) was also used to further determine the chemical composition (Figure 2n) and states of the elements of the PVP@CaO_2_ nanoparticles. As shown in Figure 2p, 287.98, 285.68, and 284.78 eV in the C 1s spectra, which correspond to C = O, C‐O and C‐C, respectively, are the characteristic bonds of the carbon functional groups in the nanoparticles. Figure 2q shows that the peaks at 532.98, 532.08, 531.58, and 531.08 eV in the high‐resolution spectra of O 1s spectra belong to Ca‐O, C = O, O‐O and C‐O, respectively. In the high‐resolution spectra of Ca 2p (Figure 2r), the peaks at 350.88 eV (Ca 2p1/2) and 347.28 eV (Ca 2p3/2) two characteristic peaks were observed indicating its presence in the free calcium state. Next, oxygen release from PVP@CaO_2_ was investigated using microelectrodes. Specifically, the PVP@CaO_2_ group reached the upper limit of dissolved oxygen (260 × 10^−6^ m) within 10 min when catalase was added, which facilitated the rapid attainment of hyperoxia and promoted wound healing. As a promising oxygen carrier, CaO_2_ nanoparticles react with water to generate hydrogen peroxide, which is decomposed into oxygen by catalase [53]. All other conditions being equal, the dissolved oxygen concentration of PVP@CaO_2_ + catalase is greater than that of PVP@CaO_2_, because catalase can quickly convert hydrogen peroxide into dissolved oxygen, thereby releasing oxygen quickly and efficiently (Figure 2m).

### 2.3 Synthesis and Characterization of CF Hydrogels

**FIGURE 3:**
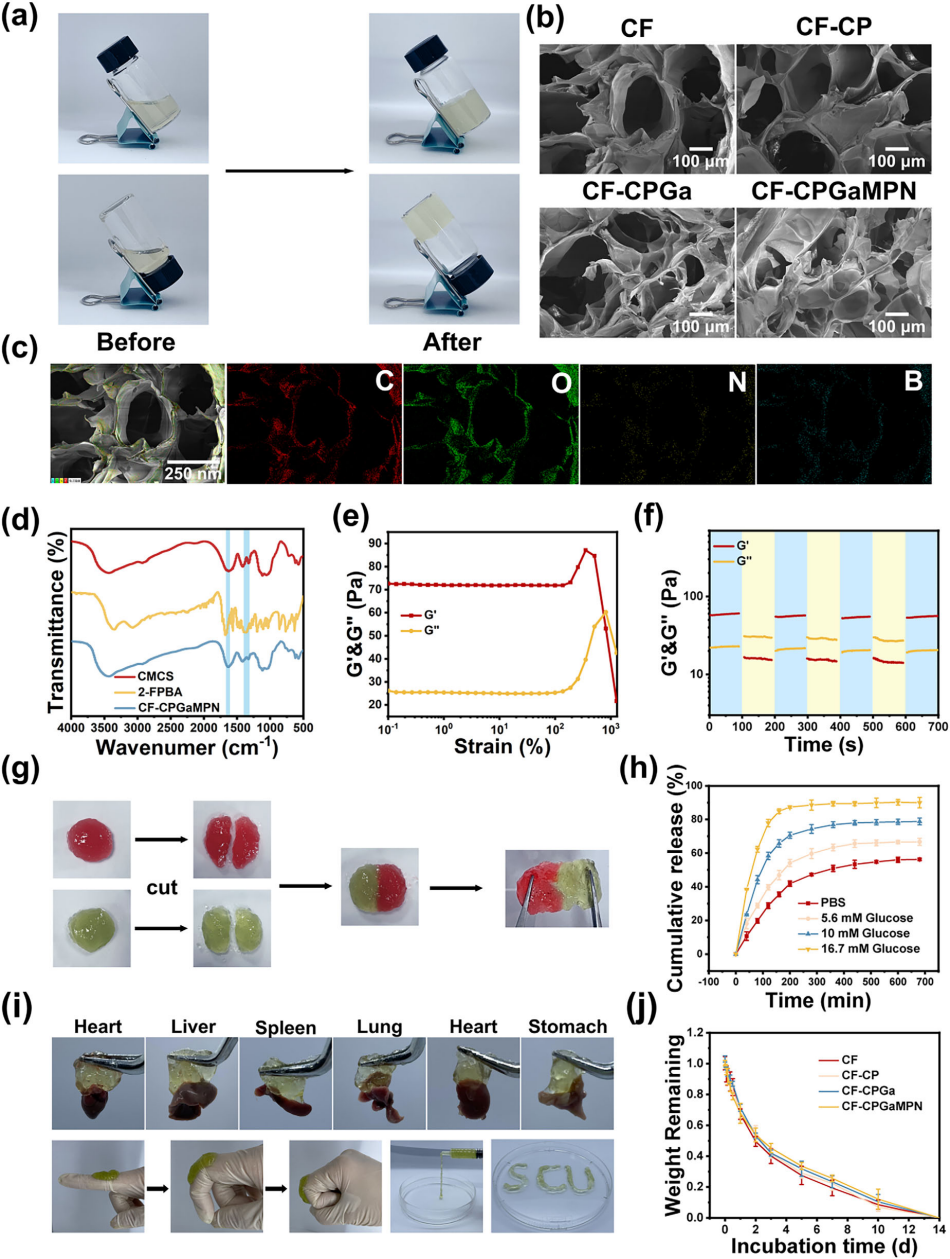
Characterization of CF hydrogels. (a) Photographs of CF hydrogels before and after gel formation. (b) SEM images of CF hydrogels. (c) Element mapping of CF hydrogels. (d) FT‐IR images of CF hydrogels. (e) Amplitude scan test curves for CF‐CPGaMPN hydrogels. (f) Dynamic rheological cycling test images of CF‐CPGaMPN hydrogels. (g) Self‐healing images of CF hydrogels. (h) The release curves of GaMPN nanoparticles in CF‐CPGaMPN hydrogel in PBS solution, 5.6 mM glucose solution, 10 mM glucose solution and 16.7 mM glucose solution. (i) Photographs of CF hydrogel adhesion to different organs and injectable properties. (j) Degradation curves for each group of CF hydrogels.

The CMCS hydrogel precursor solution was photographed and then the CF hydrogel formed after the addition of 2‐FPBA was photographed as shown in Figure 3a. The amino group on CMCS formed the first dynamic covalent cross‐linking (Schiff base bond) with the aldehyde group on 2‐FPBA. In addition, the second dynamic covalent cross‐linking (borate bond) was formed between the borate group on 2‐FPBA and the phenol group on GaMPN, which together formed the cross‐linking network of CF‐CPGaMPN hydrogels. The morphology of the CF hydrogels was first observed using SEM, and as shown in Figure 3b, all groups of CF hydrogels showed porous microstructures. In addition, the energy dispersive X‐ray spectroscopy (EDS) elemental mapping of the CF hydrogels (Figure 3c) indicated that the elements C, O, N, and B were uniformly distributed within the hydrogels. The FT‐IR spectroscopy results showed (Figure 3d) that the tensile vibration peak of the imide bond in CF hydrogel is located at 1630 cm^−1^, indicating that a Schiff base is formed between CMCS and 2‐FPBA. The boro‐oxygen bond (B‐O) moved from 1370 cm^−1^ to 1320 cm^−1^, indicating the formation of a borate ester bond between GaMPN and 2‐FPBA. Figure 3g shows the macroscopic self‐healing properties of CF hydrogels. The CF hydrogels were cut into two halves, stained separately and brought into close contact. After being left at 37°C for 5 min, the two hydrogels healed into one complete hydrogel, which did not fracture upon pulling. The good self‐healing properties of CF hydrogel prevent the potential dangers associated with hydrogel rupture during exercise, which is very beneficial in wound repair. CF hydrogels are also injectable, able to be injected through a syringe needle and molded into “SCU” letters. The injectable nature of the hydrogel allows it to fit effectively around the edges of irregular wound defects for better wound repair. Due to its abundant hydrogen bonds and flexible dynamic covalent crosslinking, CF hydrogel also exhibits strong adhesion to the fingers and to the heart, liver, spleen, lungs, and kidneys (Figure 3i). Good adhesion ensures that the hydrogel dressing will not shift during application due to movement. In addition, we also measured the oxygen release curve of the CF‐CP‐GaMPN hydrogel (Figure S2a). The CF‐CPGaMPN hydrogel can stably and continuously release oxygen, effectively alleviating the hypoxic microenvironment of diabetic infected wounds, which provides a solid foundation for its application in promoting wound healing.

### 2.4 Rheological Properties of CF Hydrogels

The rheological properties of CF hydrogels were evaluated by G′ (energy storage modulus) and G″ (loss modulus). The amplitude scan test curves of the hydrogels are shown in Figure 3e, where the G′ and G″ curves cross at 816% strain. The G″ values of the hydrogels were less than G′ up to 816% strain, indicating the formation of an elastic solid‐like gel. Dynamic rheological cycling tests were also carried out to assess the self‐healing ability of the CF hydrogels, and the results are shown in Figure 3f, the gel transitions to a sol state (G′ < G″) at a strain of 1000%, but rapidly recovers its gel structure (G′ > G″) when the strain is reduced to 0.1%. This result suggests that Schiff bonds in hydrogels break at high stresses but rapidly rebuild when the stress is unloaded. Based on these results, the hydrogel network fully recovers after damage, confirming its self‐healing ability.

### 2.5 Glucose‐Responsive Release of GaMPN

The boronate ester bonds formed between GaMPN and 2‐FPBA within the CF‐CPGaMPN hydrogel are sensitive to changes in glucose concentration [54]. In a high‐glucose environment, these boronate ester bonds become unstable as excess glucose binds to the boronic acid, leading to the release of GaMPN nanoparticles [55]. In this study, we investigated the release of GaMPN nanoparticles from the CF‐CPGaMPN hydrogel under varying glucose concentrations. The standard release curve of GaMPN nanoparticles is presented in Figure S2b, while the time‐dependent release percentage curve of GaMPN nanoparticles is shown in Figure 3h. It can be observed that the continuous release of GaMPN occurred in the CF‐CPGaMPN hydrogel, and the drug release accelerated at high glucose concentrations. Under physiological conditions (PBS), GaMPN was continuously released from the CF‐CPGaMPN hydrogel, and the cumulative release amount of GaMPN at 680 min was 56.27 ± 0.67%. At high glucose concentrations, the release rate of GaMPN in CF‐CPGaMPN hydrogel was faster. At 680 min, the release amount of GaMPN in CF‐CPGaMPN hydrogel increased to 66.70 ± 2.10% (5.6 mM Glucose), 78.80 ± 2.02% (10 mM Glucose), and 89.95 ± 3.07% (16.7 mM Glucose). The glucose‐responsive release of CF‐CPGaMPN hydrogel is beneficial for maximizing the utilization of drugs in the microenvironment of diabetic infected wounds, thereby intelligently promoting the healing of diabetic infected wounds.

### 2.6 Swelling and Degradation Properties of CF‐CPGaMPN Hydrogel

Figure S2c shows the swelling behavior of each group of CF hydrogels in a moist environment, each group of CF hydrogels initially exhibited rapid swelling behavior for 5 h. After 24 h, the swelling of each CF hydrogel formulation stabilized, with no notable differences in swelling rates observed across the groups. Hydrogel dressings can absorb moisture and tissue exudates while maintaining a moist wound environment, thereby assisting in infection control and promoting the healing of infected wounds [56].

The degradability of biomaterials is a basic requirement for their application in biomedicine. The degradation curves of each group of hydrogels in PBS are shown in Figure 3j. The results indicated that the degradation trends of each group of hydrogels were similar. The CF‐CPGaMPN hydrogel could maintain a gel state for more than 10 days in vitro, covering the critical period of wound healing.

### 2.7 Biocompatibility Evaluation of CF‐CPGaMPN Hydrogels

**FIGURE 4:**
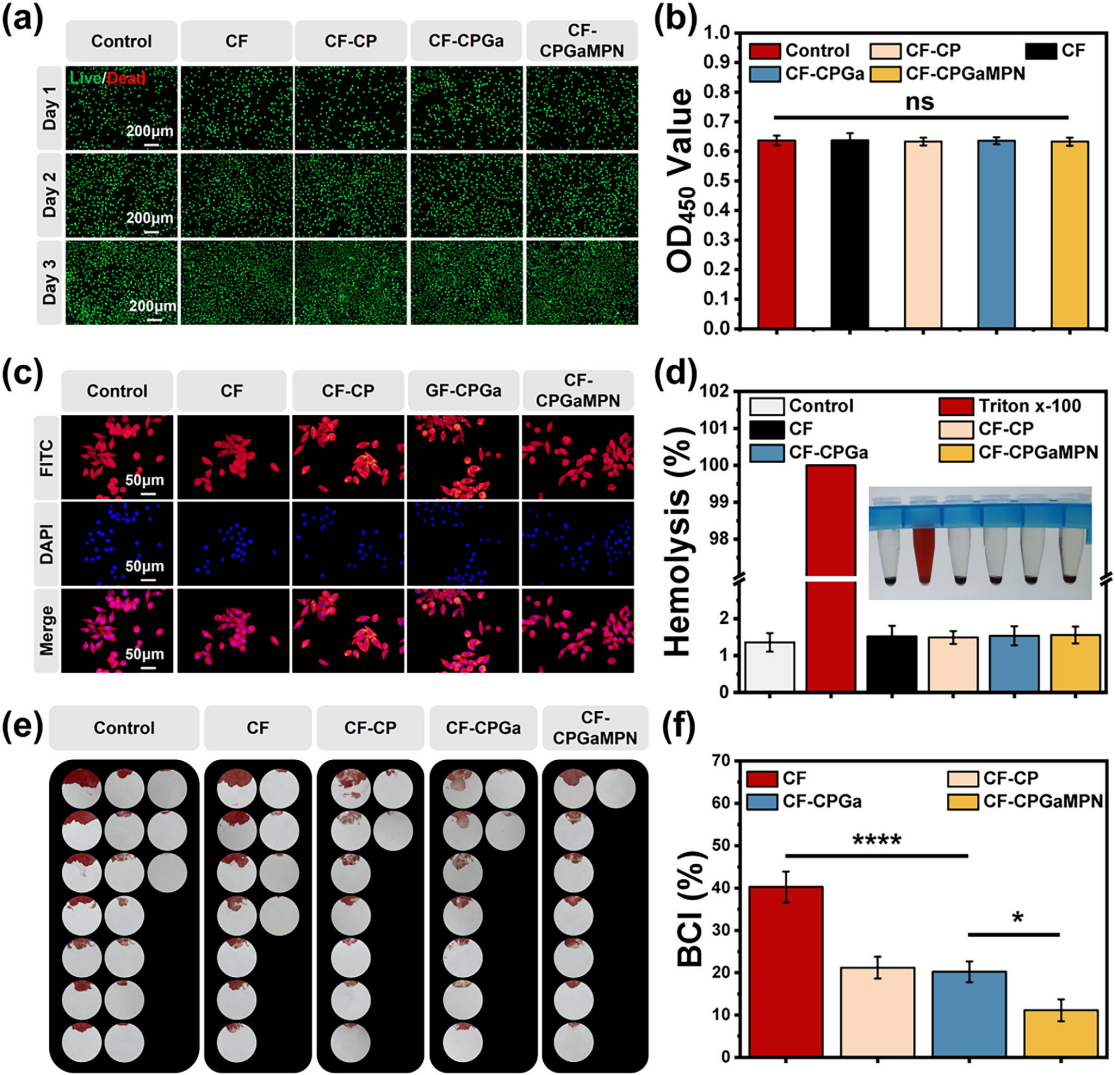
Biocompatibility, haemocompatibility and haemostatic properties of CF‐CPGaMPN hydrogels. (a) Live/dead fluorescence images of L929 cells after co‐culture with CF hydrogels. (b) OD values of L929 cells co‐cultured with CF hydrogels. (c) Fluorescence images of L929 cell morphology co‐cultured with CF hydrogels. (d) Haemolysis rate of CF hydrogels. (e) Liver haemorrhage images (filter paper changed every 15 s). (f) Coagulation index (BCI %) after CF hydrogels treatment. *p < 0.05, **p < 0.01, ***p < 0.001, ****p < 0.0001, n = 3.

Good biocompatibility is essential for the in vivo application of biomaterials. The in vitro cytotoxicity of hydrogel dressings was first assessed by Live/Dead staining images. Most L929 cells in each hydrogel group had green fluorescence and spindle‐shaped morphology, and the cell density on the third day was significantly higher than that on the first day (Figure 4a). The results of CCK‐8 assay were consistent with those of cell viability and death staining. No significant cytotoxicity was found in L929 Fibroblasts after 24 h of co‐incubation with each group of hydrogel dressings, and the cell viability was not significantly different from that of the blank group (Figure 4b). We also observed the cell morphology of L929 cells after co‐culture with each group of CF hydrogel by cytoskeleton and nucleus staining (Figure 4c). After 3 days of co‐culture, the cells in both the control and hydrogel groups were able to adhere and grow, and the cells in each group had a uniformly stretched morphology. The above results indicated that all groups of CF hydrogels had excellent cytocompatibility.

In addition, haemocompatibility was estimated using an in vitro haemolysis test. PBS and Triton x‐100 served as the negative and positive controls, respectively. All hydrogel samples exhibited a light red coloration, comparable to that observed in the PBS group. The Triton x‐100 group was bright red and had the highest rate of haemolysis, whereas all hydrogel groups showed a fairly low rate of haemolysis (<2%), indicating good haemocompatibility (Figure 4d).

To investigate the in vivo haemostatic ability of CF‐CPGaMPN hydrogel, a mouse liver haemorrhage model was established. When CF‐CPGaMPN hydrogel was applied to the liver bleeding site, there was a small amount of bleeding on the filter paper and the hemostasis was rapid, whereas the control mice without hydrogel had more bleeding and slow hemostasis (Figure 4e). The dynamic whole blood‐clotting assay is frequently employed to assess the clotting capacity of hemostatic materials. A lower blood clotting index (BCI) corresponds to a more rapid coagulation rate [57]. The BCI values of all hydrogel groups were significantly lower than those of the control group, and the coagulation ability of CF‐CP and CF‐CPGa hydrogels was stronger than that of CF hydrogels. Among them, CF‐CPGaMPN hydrogel group had the strongest coagulation ability (Figure 4f). CF‐CPGaMPN hydrogel promotes hemostasis because the positive charge on CMCS attracts erythrocytes through electrostatic action [58], and Ca^2+^ released by the hydrogels acts as a cofactor in several coagulation cascades and platelet activation [59]. In addition to this, TA also has excellent coagulation activity due to its ability to react with blood proteins (serum albumin, globulin and coagulation factors) [60].

### 2.8 In Vitro Antimicrobial Properties of CF‐CPGaMPN Hydrogels

**FIGURE 5:**
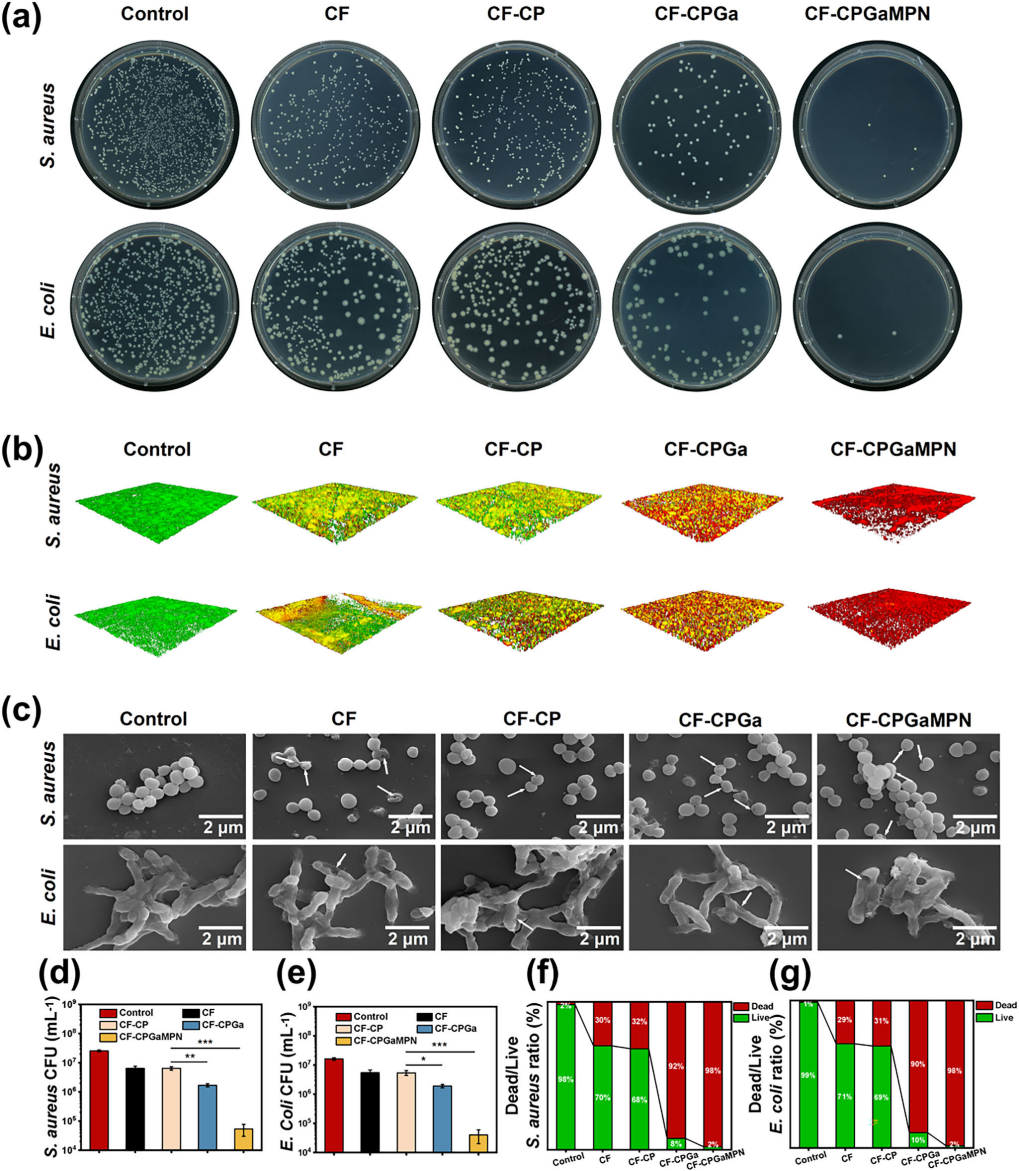
In vitro antimicrobial efficacy of CF‐CPGaMPN hydrogels. (a) CFU images of *E. coli* and *S. aureus* after different treatments. (b) 3D live/dead fluorescence staining of *E. coli* and *S. aureus* biofilms after treatment, with green and red fluorescence indicating live and dead bacteria, respectively. (c) SEM images of *S. aureus* and *E. coli* biofilm, the damage and degeneration of the cell membrane are marked with white arrows. (d) Antimicrobial efficiency of each group of CF‐CPGaMPN hydrogels against *S. aureus*. (e) Antimicrobial efficiency of each group of CF‐CPGaMPN hydrogels against *E. coli*. (f) Corresponding proportions of live and dead *S. aureus* in biofilms. (g) Corresponding proportions of live and dead *E. coli* in biofilms. *p < 0.05, **p < 0.01, ***p < 0.001, ****p < 0.0001, and n = 3.

Diabetic wounds often present a complex microenvironment that is highly vulnerable to bacterial colonization [61]. Hence, hydrogel‐based wound dressings should exhibit adequate tissue adhesion to minimize secondary contamination, as well as demonstrate inherent antimicrobial activity to prevent external bacterial invasion. The antimicrobial properties of CF‐CPGaMPN hydrogels were investigated by co‐culturing them with Staphylococcus aureus (*S. aureus*) and Escherichia coli (*E. coli*). As depicted in Figure 5a, colony forming unit (CFU) assays revealed the relative viability of *S. aureus* and *E. coli* following various treatments. And the statistical results (Figure 5d,e) indicated that CF has a certain antibacterial effect by itself compared to the control group, which may attribute to the cationic antibacterial effect of CMCS. Similar antimicrobial effects could be observed on CF‐CP, verifying that the incorporation of PVP@CaO_2_ and Catalase did not alter the CF hydrogel efficacy. The introduction of Ga^3+^ and GaMPN enhanced the antimicrobial effect of CF hydrogels in the CF‐CPGa and CF‐CPGaMPN groups, and CF‐CPGaMPN had the strongest antibacterial activity. Subsequent live/dead assays using 3D confocal laser scanning microscopy (CLSM) yielded similar results (Figure 5b). The control group exhibited strong green fluorescence, indicative of predominantly viable bacteria. There was a large amount of green fluorescence and a small amount of red fluorescence (dead bacteria) in the CF and CF‐CP groups, with the CF‐CPGa group having significantly more dead bacteria and the CF‐CPGaMPN group having almost no detectable live bacteria. Quantification of the live/dead bacteria ratio further showed that the CF‐CPGaMPN group had the highest percentage of dead *S. aureus* and *E. coli* and the lowest percentage of live *S. aureus* and *E. coli* (Figure 5f,g). The bactericidal activity of CF‐CPGaMPN was significantly higher than that of Ga^3+^, which may be attributed to the enhanced uptake of Ga^3+^ by bacteria when GaMPN nanoparticles are formed. In addition, the destructive effects of each group of hydrogels on the biofilms of Escherichia coli and Staphylococcus aureus were studied by crystal violet staining. As shown in Figure S3a, the staining depth of the CF‐CPGaMPN hydrogel biofilm became shallower, indicating that the biofilm was destroyed. The absorbance measured at 590 nm was used to quantitatively evaluate the bacterial activity (Figure S3b,c). The results showed that all the groups of hydrogels could destroy the bacterial biofilms, among which the CF‐CPGaMPN hydrogel had the strongest ability to destroy the bacterial biofilm. Bacterial morphology following each treatment was further examined using SEM. As presented in Figure 5c, untreated *S. aureus* and *E. coli* cells displayed their characteristic spherical and rod‐shaped forms, respectively, exhibiting smooth and intact cell walls. In contrast, in the CF‐CPGaMPN group, some bacterial cell membranes were ruptured, (white arrows in Figure 5c), and some bacterial cell membranes were intact. This observation aligns with the proposed dual antibacterial mechanism of the CF‐CPGaMPN hydrogel: First, the cationic CMCS component can bind to the negatively charged bacterial membranes via electrostatic interactions, which disrupts the bacterial osmotic balance and leads to membrane rupture and bacterial death [62]. This explains the observed membrane damage. Second, the antimicrobial mechanism of GaMPN does not primarily rely on membrane disruption. Instead, it functions via a “Trojan Horse” strategy. Through the bacterial heme‐acquisition system, GaMPN is mistaken for heme (iron source) and is actively transported into the cytoplasm. Once inside, Ga^3^^+^ cannot undergo redox cycling like Fe^3^^+^, thereby interrupting critical enzymatic metabolism and inducing bactericidal effects without causing discernible morphological damage to the bacterial membrane [63, 64]. This mechanism accounts for the presence of intact but non‐viable bacteria in the SEM images. The coexistence of ruptured and intact bacterial thus provides complementary evidence for the synergistic action of the two components.

In addition, we also conducted an in vitro methicillin‐resistant Staphylococcus aureus (*MRSA*) antibacterial experiment using the CF‐CPGaMPN hydrogel, and the results were consistent with those for *S. aureus* and *E. coli*. Among all the tested hydrogel groups, the CF‐CPGaMPN hydrogel exhibited the strongest and most significant antibacterial activity against *MRSA*. The CFU count results showed that it could significantly reduce the number of viable bacteria (Figure S4a,c), and the live/dead staining images also clearly demonstrated that the vast majority of *MRSA* bacteria were killed (showing red fluorescence) (Figure S4b,d). These supplementary data further confirm that the CF‐CPGaMPN hydrogel is not only effective against standard laboratory strains, but also possesses strong and broad‐spectrum antibacterial capabilities against clinically relevant drug‐resistant pathogens such as *MRSA*. This enhances the clinical relevance and persuasiveness of our research results.

### 2.9 The Effect of CF‐CPGaMPN Hydrogel on the Healing of Infected Wounds in a Diabetic Mouse Model

**FIGURE 6:**
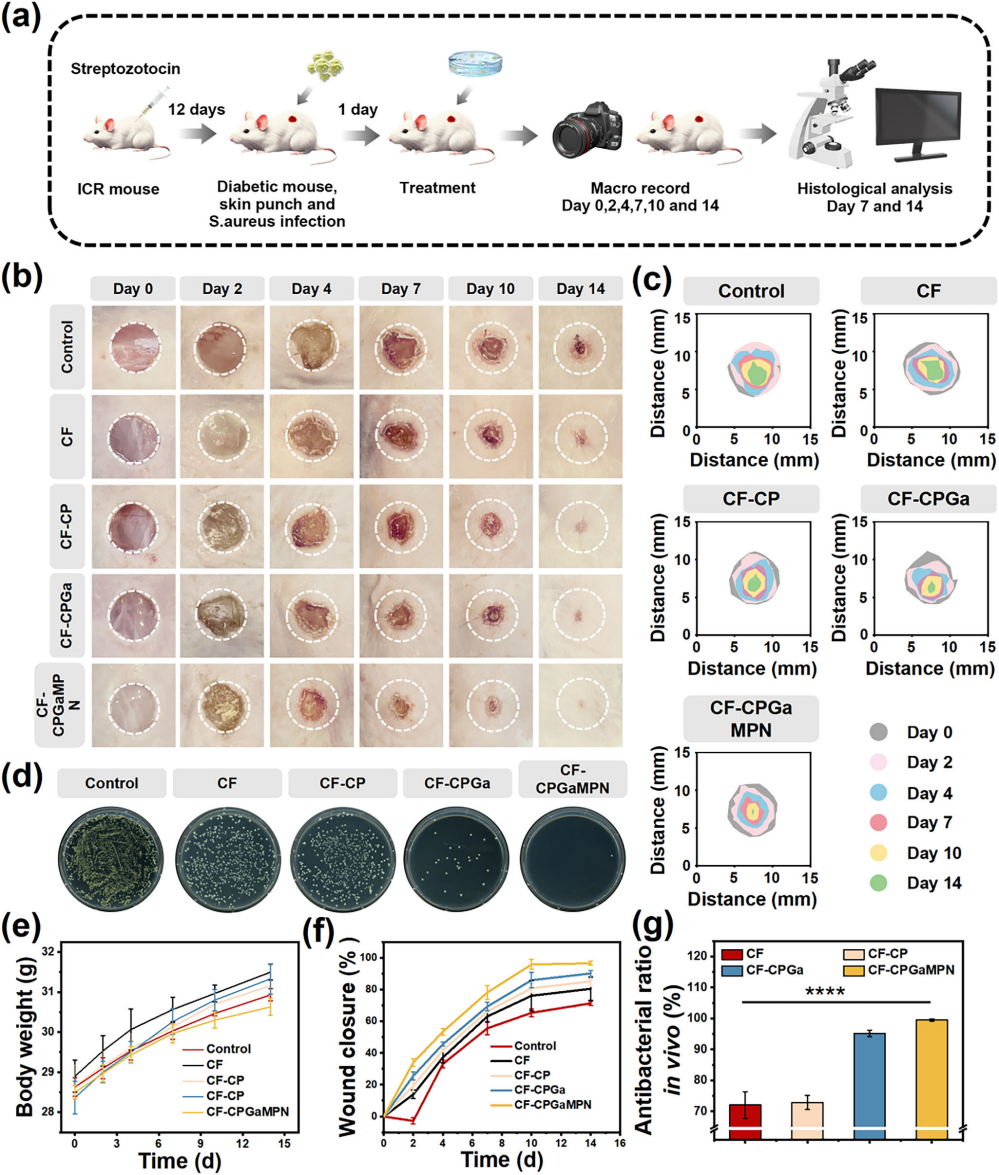
In vivo antibacterial effect of CF‐CPGaMPN hydrogels. (a) The process of in vivo animal experiments. (b) Representative images of skin wounds from each group between day 1 and day 14. (c) Representative changes of wound closure over time. (d) CFU were visualized after 2 days of treatment. (e) Weight change over time in mice after surgery. (f) Changes in the size of the wound over time. (g) Antibacterial ratio in vivo. *p < 0.05, **p < 0.01, ***p < 0.001, ****p < 0.0001, and n = 3.

A streptozotocin‐induced diabetic mouse model was utilized to assess the in vivo efficacy of the hydrogel in promoting the closure of infected diabetic wounds (Figure 6a). As shown in Figure 6b, there was a gradual decrease in the remaining wound area over the course of the treatment, with the hydrogel‐treated wounds being significantly smaller than the controls at the same healing time point. Notably, wound expansion occurred in the control group—a phenomenon frequently observed in chronic diabetic wounds and attributed to excessive inflammatory response (Figure 6c). After 14 days of healing, the wound healing rates were approximately 71.39 ± 1.33%, 80.52 ± 2.31%, 85.2 ± 3.05%, 90.19 ± 0.91%, and 96.61 ± 1.31% in the control group, CF hydrogel, CF‐CP hydrogel, CF‐CPGa hydrogel, and CF‐CPGaMPN hydrogel, respectively (Figure 6f). Results indicated that wounds treated with CF‐CPGaMPN hydrogel exhibited nearly complete closure, whereas those in the control group remained unhealed. The CF‐CPGaMPN hydrogel group demonstrated the most rapid healing rate among all groups. Figure 6d shows the CFU of *S. aureus* in the wound after 2 days of treatment. Consistent with its in vitro antimicrobial activity, the CF‐CPGaMPN hydrogel treatment reduced the number of viable colonies by approximately 99% compared to the control group (Figure 6g). We also monitored body weight changes in the mice after surgery and observed a stable weight increase in the mice from all groups, suggesting that all groups of CF hydrogel were not significantly toxic to the mice (Figure 6e).

### 2.10 Histological Staining

**FIGURE 7:**
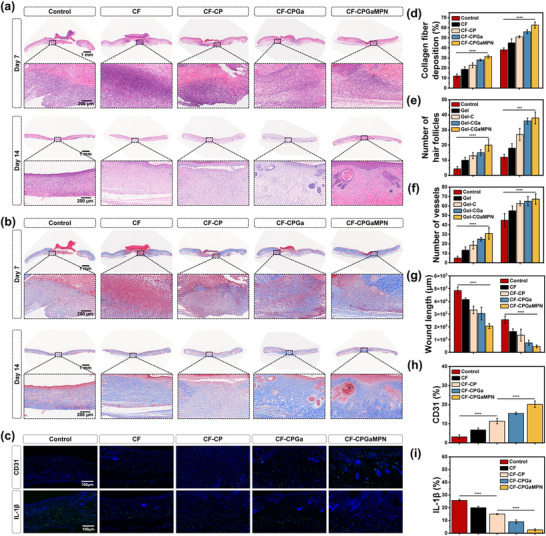
In vivo studies demonstrate the efficacy of CF‐CPGaMPN hydrogels in enhancing the healing of infected skin tissue. (a) H&E staining of tissue sections from wound sites on day 7 and day 14. (b) Masson's trichrome staining of wound tissue sections obtained at day 7 and day 14. (c) Immunofluorescence images of CD31 and IL‐1β expressed in wound tissue sections. (d) Quantitative data of the number of collagen fibers deposited. (e) Quantitative data of the number of hair follicles. (f) Quantitative data of the number of vessels. (g) Quantitative data of wound length. (h) Quantitative data of the percentage of CD31. (i) Quantitative data of the percentage of IL‐1β. *p < 0.05, **p < 0.01, ***p < 0.001, ****p < 0.0001, and n = 3.

To histologically assess the wound healing promoted by CF‐CPGaMPN hydrogel, skin tissue samples from the wound sites were subjected to Hematoxylin and Eosin (H&E) staining, Masson's trichrome staining, and immunofluorescence staining. According to H&E staining, treatment with CF hydrogel enhanced the regeneration of both epidermal and subepithelial structures across all groups relative to the control (Figure 7a). By day 14, H&E and Masson's trichrome staining revealed that the newly formed skin tissues in the CF‐CPGaMPN group more closely resembled normal skin in terms of hair follicle formation, skin appendages, and epidermal thickness (Figure 7a,b). By calculating and analyzing the collagen fiber deposition (Figure 7d), the number of hair follicles (Figure 7e), the number of blood vessels (Figure 7f), and the length of the wound (Figure 7g) in each group, the CF hydrogels in each group were able to promote the wound healing process. Among them, CF‐CP hydrogel promoted wound healing more than CF hydrogel probably because the oxygen released from CF‐CP hydrogel improved the hypoxic microenvironment of chronic diabetic infected wounds, which promoted the wound healing process. CF‐CPGaMPN hydrogel promoted wound healing the most obviously, due to the strong antimicrobial efficacy of GaMPN nanoparticles compared to Ga^3+^, which could eliminate wound infection and further promote chronic diabetic wound healing.

In addition, immunofluorescence staining was performed to observe the angiogenesis and inflammatory response around the wound. The CF‐CPGaMPN hydrogel group showed increased CD31 expression levels versus the control group (Figure 7c,h), indicating its potential angiogenic properties mediated through CD31 upregulation. IL‐1β is a marker of tissue inflammatory response, and, as shown in Figure 7c,i, the fluorescence intensity of IL‐1β in the CF‐CPGaMPN hydrogel was lower than that of the control group, suggesting that CF‐CPGaMPN hydrogel attenuated the degree of inflammatory response in the wounds of mice. The above results indicated that CF‐CPGaMPN hydrogel effectively accelerated the repair and regeneration of diabetic infected wounds by modulating the inflammatory response and promoting vascular regeneration.

Furthermore, we also conducted further animal experiments using *MRSA*. Through H&E staining and Masson's trichrome staining analysis of the wound tissue, we found that compared to the control group, the treated wounds in the CF‐CPGaMPN group showed significantly enhanced epidermal regeneration and subepithelial structure remodeling. The skin tissue structure regenerated by the CF‐CPGaMPN group was closest to the normal skin morphology, and exhibited the best repair effect at the histological level, outperforming the Biatain Alginate Ag group. The supplementary experimental results (Figure S5) indicated that in more challenging environments of *MRSA* infection, our CF‐CPGaMPN hydrogel treatment group also demonstrated significant advantages.

We also examined the in vivo biocompatibility of each group of CF hydrogels via H&E staining of mouse hearts, livers, spleens, lungs and kidneys, and the results showed the good in vivo biocompatibility of each group of CF hydrogels (Figures S6 and S7). The blood biochemical tests showed that all values of liver and kidney functions were within the normal range, with no statistical difference between groups (Figure S8).

### 2.11 Single‐Cell Mapping Reveals Heterogeneity of Diabetic Microenvironment Regulated by CF‐CPGaMPN Hydrogels

**FIGURE 8:**
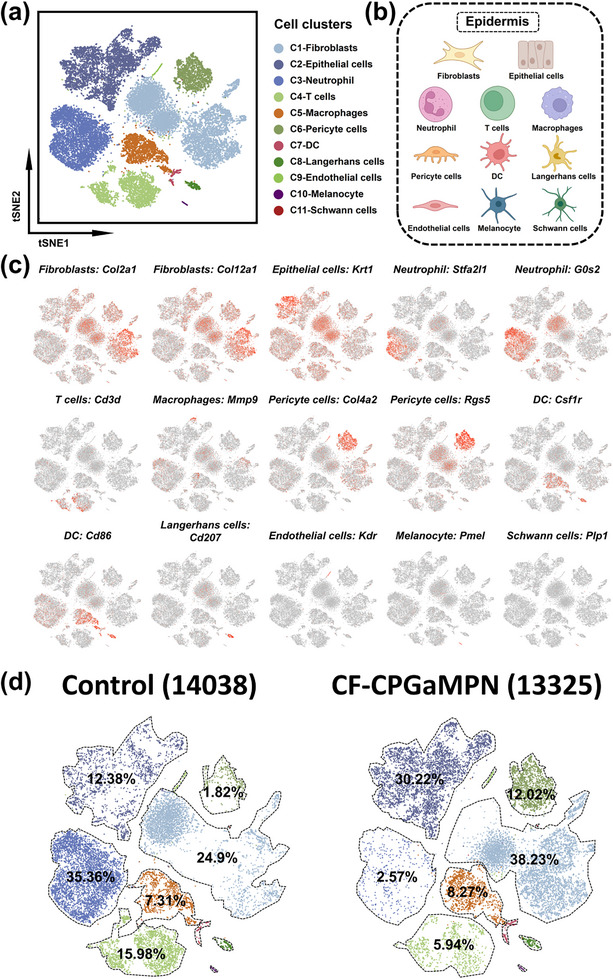
The scRNA‐seq analysis reveals cellular heterogeneity on day 7 wounds. (a) Cellular heterogeneity was evident in the t‐SNE visualization, which distinguished 11 discrete cell clusters, each marked by a distinct color. The general identity of every cluster is annotated on the right. (b) Wound schematic showing cellular repertoire. (c) Feature plots illustrate the expression distribution of chosen cluster‐specific genes. Cells exhibiting the highest expression are shown in red. (d) The distribution and proportions of the 11 clusters in the control and CF‐CPGaMPN hydrogel groups were observed by t‐SNE projection; each cluster is color‐coded, and the proportions of cells in each cluster are labeled in the figure.

To understand the underlying mechanisms of how CF‐CPGaMPN hydrogel improves diabetic wound healing, we performed scRNA‐seq to explore the molecular features by comparing the gene expression patterns between Control and CF‐CPGaMPN groups. First, we identified the cell populations around the wound as the first‐level primary cell category and classified 11 cell populations (Figure 8a). Figure 8b summarizes the cell types identified, and heat and characteristic maps for each cluster are described in Figure S9a, Figure 8c and Figure S9b. Among these cell clusters, Fibroblasts, Epithelial cells and Neutrophils had the highest proportions, accounting for ≈31.75%, ≈21.53% and ≈18.54% of all cells, respectively. Cluster C1 cells are classified as Fibroblasts, which are highly expressive of Col2a1 and Col12a1. Cluster C2 enriched Epithelial cell markers, including Krt1. Cluster C3 cells are classified as Neutrophils, whose marker genes are G0S2 and Stfa2l1. The expression level of Cd3d was used to determine T cells (Cluster C4, ≈10.83%) and Mmp9 was used to identify Macrophages (Clster C5, ≈7.8%). Peripheral cells (cluster C6, ≈7.05%) showed increased levels of Col4a2 and Rgs5, Dendritic Cells (DC) cells (cluster C7, ≈0.85%) expressed high levels of Csf1r and Cd86, and Langerhans cells (cluster C8, ≈0.85%) expressed elevated levels of Cd207. Cluster C9 cells were identified as Endothelial cells (≈0.46%) expressing high levels of Kdr, Cdh5 and Tie1, C10 as Melanocytes (≈0.27%) expressing high levels of Pmel, Tyr, Mlana, Npy and Pax3, and C11 as Schwann cells (≈0.07%) expressing high levels of Plp1.

We found that the proportion of Fibroblasts (Cluster C1) and Epithelial cells (Cluster C2) was higher in the CF‐CPGaMPN hydrogel group, but the proportion of Neutrophils (Cluster C3) was higher in the Control group. Specifically, the proportions of Fibroblasts were 38.23% and 24.93% in the CF‐CPGaMPN hydrogel group and Control group, respectively; the proportions of Epithelial cells were 30.22% and 12.38%, respectively; and the proportions of Neutrophils were 2.57% and 35.36%, respectively (Figure 8d). These results indicated that the Control group had more inflammatory cells (Neutrophils) and the CF‐CPGaMPN hydrogel group had more healing cells (Fibroblasts, Epithelial cells) after 7 days of treatment. The CF‐CPGaMPN hydrogel group affects cellular infiltration during diabetic‐infected wound healing, with the ability to regulate Fibroblasts, Epithelial cells and Neutrophils which reduces periwound inflammation and creates a balanced immune microenvironment.

### 2.12 CF‐CPGaMPN Hydrogel Recruits Slpi+ Neutrophils and Suppresses S100a9+ Neutrophils in Diabetic Wounds

Throughout the repair process of diabetic wound repair, interactions between different cell types provide coordination of individual events. Wound healing begins with an early phase dominated by Neutrophil‐mediated inflammation. The contribution of Neutrophils to wounding depends largely on their heterogeneity, which is regulated by local cues in the microenvironment. In the case of diabetes mellitus, Neutrophils may be in a state of hyperinflammation or not properly removed from the injured tissue, leading to persistent and nonhealing wounds [65]. The broad functions of Neutrophils include phagocytosis, degranulation, and the formation and release of NETs, which are network structures composed of chromatin filaments modified by granulin [66, 67]. In the case of normal wounds, low concentrations of NETs promote proliferation of Epidermal keratinocytes [68]. However, overproduction of NETs in diabetic wounds leads to delayed wound healing [69]. Unlike previous necrosis and apoptosis, NETs formation accompanied by Neutrophil death is called suicidal NETosis and takes ≈1–4 h. NETosis is favored by the oxidative stress environment and inflammatory factors produced by inflammatory cells in the diabetic environment [69].

**FIGURE 9:**
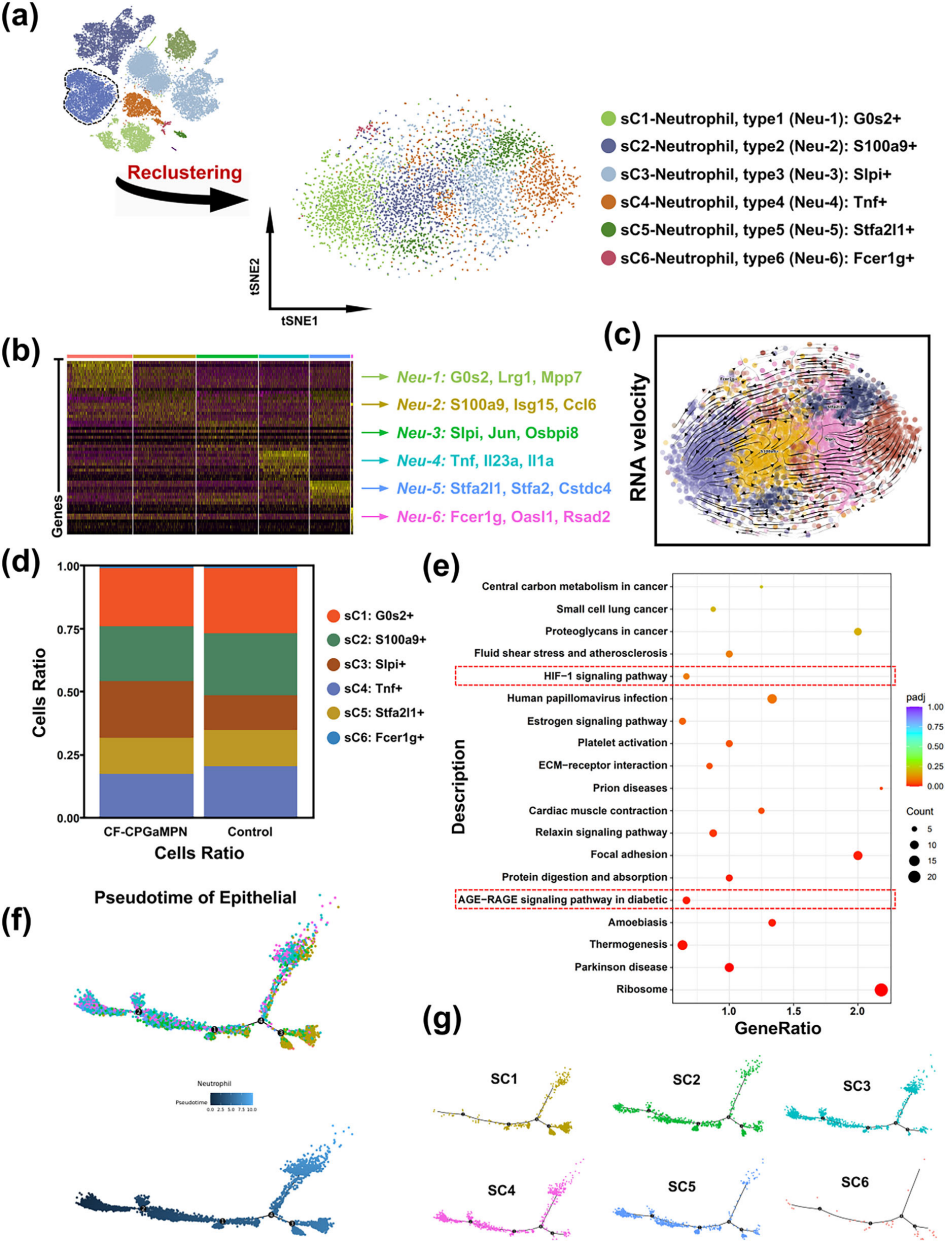
Identification of Neutrophil subclusters in hydrogels‐regulated microenvironment. (a) Neutrophils underwent re‐clustering in the t‐SNE projection, resulting in six discernible subpopulations that were color‐coded. Identities of these subclusters are annotated in the figure. (b) Differentially expressed genes within neutrophil subsets are visualized in the heatmap. Representative genes for each cluster appear color‐coded on the right. (c) RNA velocity of Neutrophils; color coded by clusters. (d) Histograms of the proportion of Neutrophil subclusters in the control and CF‐CPGaMPN hydrogel groups. (e) KEGG enrichment analysis of descending genes in CF‐CPGaMPN hydrogel sets. (f,g) Pseudotime lineage trajectory analysis demonstrating the relationships of subclusters among Neutrophils (f), color coded by subclusters (g).

To elucidate the heterogeneity of Neutrophils in the microenvironment of diabetic infected wounds regulated by CF‐CPGaMPN hydrogels, Neutrophils were repooled for subgroup categorization and identified into six subclusters, sC1‐sC6, in t‐SNE (Figure 9a). Representative marker genes for each subgroup are listed in the heatmap (Figure 9b). Cluster sC1 (G0s2+) included G0s2, Lrg1, and Mpp7. cluster sC2 (S100a9+) was enriched for S100a9, Isg15, and Ccl6. cluster sC3 (Slpi+) included Slpi, Jun, and Osbpi8. sC4 cells (Tnf+) expressed Tnf, ll23a, and ll1a. sC5 (Stfa2l1+) expressed Stfa2l1, Stfa2, and Cstdc4. cluster sC6 (Fcer1g+) representative markers are Fcer1g, Oasl1, and Rsad2.

S100a9 is recognized as a marker of NETs release and Neutrophil activation [70]. Secreted leukocyte protease inhibitor (Slpi), an inhibitor of Neutrophil elastase and nuclear factor κB, plays a role in anti‐NETs by inhibiting the formation and blocking the release of NETs [71]. We found a lower percentage of S100a9+ Neutrophils (sC1) and a higher percentage of Slpi+ Neutrophils (sC1) in diabetic wounds in the CF‐CPGaMPN hydrogel‐treated group compared to the Control group (Figure 9d). We next performed RNA velocity (Figure 9c) and trajectory (Figure 9f,g) analyses, revealing that the pseudotemporal distributions of the S100a9 subcluster and the Slpi subcluster were located in the more anterior position. These results suggest that CF‐CPGaMPN hydrogel plays an important role in reducing NETs and a positive role in inhibiting NETs‐induced long‐term diabetic wound repair.

In diabetic wounds, advanced glycosylation end products (AGEs) tend to build up. The interaction between AGEs and their receptors (RAGE) plays a role in the development of dermatopathic complications associated with diabetes. Studies show that inhibition of AGE‐RAGE signaling enhances wound healing in diabetic mouse models [72]. Hypoxia leads to significant cellular stress and inability to carry out important biological processes, and thus hypoxia‐inducible factor‐1 (HIF‐ 1) is activated to match O_2_ supply to the metabolic and energy demands of the cells [73]. KEGG enrichment analysis of Neutrophils in diabetic infected wounds in the CF‐CPGaMPN hydrogel group showed downregulation of the AGE‐RAGE signaling pathway associated with diabetic complications and the HIF‐1 signaling pathway associated with cellular hypoxia (Figure 9e). These results suggest that CF‐CPGaMPN hydrogel not only has a mitigating effect on cellular hypoxia, but also reduces the chance of diabetes‐related complications. In conclusion, CF‐CPGaMPN hydrogel has a positive effect in inhibiting long‐term diabetic wounds induced by NETs.

### 2.13 CF‐CPGaMPN Hydrogel Recruits Ccl3+ Fibroblasts and Suppresses Vcam1+ Fibroblasts in Diabetic Wounds

In the middle and late stages of wound healing, cellular interactions are dominated by Epidermal cell – Fibroblast interactions, which gradually transform the microenvironment from inflammatory to granulation tissue [74]. Fibroblasts are essential for improving tissue healing and maintaining tissue homeostasis [75]. Therefore, we investigated the regulatory effect of CF‐CPGaMPN hydrogel on the phenotype of Fibroblasts in wounds. Fibroblasts were segregated into eight different functional subpopulations sC1 to sC8 (Figure S10a). They were differently proportioned in the Control and CF‐CPGaMPN hydrogel groups (Figure S10d). The marker genes for each subcluster are shown as heatmaps (Figure S10b). Specifically, Vcam1, Col14a1, and Col1a2 were elevated marker genes in cluster sC1 (Vcam1+); cluster sC2 (Ccl3+) included Ccl3, Ccl4, and Cxcl2. sC3 (Lars2+) included Lars2, Camk1d, and Gphn. sC4 cells (Col7a1+) expressed Col7a1, Tnn and Crabp1. sC5 (Lsmem1+) expresses Lsmem1, Cd3e, and Hp. sC6 (Trps1+) representative markers are Trps1, Robo2, and Enpp2. sC7 (Penk+) highly expresses Penk, Nos2, and Thbd. sC8 cells (Igfbp5+) expressed Igfbp5, Csmd1 and Pde1c.

During diabetic wound healing, Ccl3 stimulates the proliferation of Fibroblasts, Keratinocytes, and Endothelial cells [76]. Vascular cell adhesion molecule‐1 (Vcam1) is an important adhesion molecule involved in the adhesion process of white blood cells to vascular endothelial cells, thereby triggering inflammatory response [77]. However, in refractory diabetic wounds, due to long‐term high glucose status, metabolic disorders and other factors, vascular endothelial cells are damaged, Vcam1 expression is increased, and leukocyte adhesion is increased, thus triggering sustained inflammatory response [78]. Therefore, reducing the expression level of Vcam1 may help accelerate the healing of diabetic wounds. The experimental results showed a higher proportion of Ccl3+ Fibroblasts (sC2) and a lower proportion of Vcam1+ Fibroblasts (sC1) in the CF‐CPGaMPN hydrogel group compared with the Control group (Figure S10d). These results suggest that CF‐CPGaMPN hydrogel increased the proportion of Fibroblast Ccl3+ subclusters and decreased the proportion of Fibroblast Vcam1+ subclusters to promote diabetic wound repair.

Despite observed heterogeneity in wound fibroblasts via t‐SNE analysis, it remained unclear whether these cells followed a common differentiation trajectory. We therefore performed RNA velocity (Figure S10c) and trajectory (Figure S10f,g) analyses. Fibroblasts from different subpopulations were widely distributed on the pseudo‐timeline, with Vcam1+ Fibroblasts predominantly occupying the beginning of the trajectory and Ccl3+ Fibroblasts predominantly occupying the end of the trajectory (Figure S10g), suggesting that the CF‐CPGaMPN hydrogel has the potential to promote the whole process of diabetic wound healing.

It has been reported that Notch signaling is activated by hyperglycemia and transduction is increased in cells from diabetic patients [79]. Studies have indicated that stimulating the Notch signaling pathway accelerates angiogenesis and facilitates wound healing in non‐diabetic mice. Conversely, under diabetic conditions, wound repair is improved by suppressing Notch signaling [79]. STZ‐induced overactivation of the Notch signaling pathway in mice [80], inhibition of Notch signaling and can improve wound healing in diabetic mice [81]. Based on the KEGG enrichment analysis of the CF‐CPGaMPN hydrogel set, Fibroblast Notch pathway activity was down‐regulated (Figure S10e), suggesting that CF‐CPGaMPN hydrogel could promote diabetic wound healing by inhibiting Notch signaling. Taken together, these results further suggest that CF‐CPGaMPN hydrogel has the potential to promote diabetic infected wound repair by affecting Fibroblasts in the wound.

### 2.14 CF‐CPGaMPN Hydrogel Recruits K16+ Epidermal Cells and Suppresses Mki67+ Epidermal Cells in Diabetic Wounds

During wound healing, the migration and differentiation of epidermal cells are essential for the regeneration of the epidermis [82]. Next, we investigated the modulatory effect of CF‐CPGaMPN hydrogel on the phenotype of Epidermal cells in diabetic wounds. Epidermal cells were segregated into eight different functional subpopulations sC1 to sC8 (Figure S11a). Their proportions were different in the Control and CF‐CPGaMPN hydrogel groups (Figure S11d). The marker genes for each subcluster are shown as heatmaps (Figure S11b). Specifically, Krt16, Krt10, and Krt1 were elevated marker genes in cluster sC1 (Krt16+); cluster sC2 (Sox6+) included Sox6, Fcgbp, and Sema5a. sC3 (Krt79+) included Krt79, Arhgap24, and Fst. sC4 cells (Mki67+) expressed Mki67, Hmgb2 and Top2a. sC5 (Gpc6+) expressed Gpc6, Pdzrn3 and Hmcn1. sC6 (Mmp9+) representative markers are Mmp9, Mmp13 and Il24. sC7 (Ccl3+) highly expressed Ccl3, Acod1 and Cd14. sC8 cells (Ikzf3+) expressed Ikzf3 Camk4 and Tox.

Mki67 is a proliferative marker in continuous hypoxic environments, and Mki67 expression is increased in prolonged hypoxic environments [83]. The experimental results showed that the proportion of Mki67+ Epidermal cells (sC4) in diabetic infected wounds was lower in the CF‐CPGaMPN hydrogel group compared to the Control group (Figure S11d), suggesting that the CF‐CPGaMPN hydrogel group could relieve the hypoxic environment in diabetic wounds. The proportion of K16+ Epidermal cells (sC3) in diabetic infected wounds was higher in the CF‐CPGaMPN hydrogel group (Figure S11d), and K16 was rapidly expressed after tissue injury and was arranged along the perinuclear region in the ultrastructure, promoting the onset of proliferation and migration behaviors [84] and further promoting tissue regeneration [85]. Taken together, these results suggest that CF‐CPGaMPN hydrogel reduced the proportion of Mki67+ subclusters of Epidermal cells and increased the proportion of K16+ subclusters of Epidermal cells in diabetic infected wounds, with the potential to alleviate the hypoxic environment of diabetic wounds and promote tissue regeneration.

We next performed RNA velocity (Figure S11c) and trajectory (Figure S11f,g) analyses, and Epidermal cells from different subpopulations were widely distributed on the pseudotimeline, with the pseudotimeline of the Mki67+ subcluster located in the middle of the range and the pseudotimeline of the K16+ subcluster located throughout the wound healing process. This suggests that CF‐CPGaMPN hydrogel has the potential to alleviate hypoxia and promote healing in diabetic infected wounds. KEGG enrichment analysis of Epidermal cells in the CF‐CPGaMPN hydrogel group also showed the downregulation of the Notch signaling pathway (Figure S11e), and the results further support the ability of CF‐CPGaMPN hydrogel to promote diabetic wound healing.

### 2.15 CF‐CPGaMPN Hydrogel Promotes Multiple Cellular Interactions in Diabetic Wound Healing

Interactions between cells in diabetic infected wounds may affect the wound's ability to repair. We performed CellChat to analyze the intercellular communication patterns among Neutrophils, Fibroblasts and Epithelial cells. The two circle plots show the overall interaction strength of ligand receptors in the Control and CF‐CPGaMPN hydrogel groups, respectively (Figure S9c). The results showed that the intercellular communication strength in the CF‐CPGaMPN hydrogel group was significantly greater than that in the Control group. A denser communication network between Neutrophils, Fibroblasts, and Epidermal cells was observed in the CF‐CPGaMPN hydrogel group, suggesting that Neutrophil‐Fibroblast‐Epidermal cell interaction signaling is important in diabetic infected wound healing. In conclusion, these data summarize our observations at the single‐cell level and further support that CF‐CPGaMPN hydrogel can downregulate the formation of NETs and alleviate hypoxia in diabetic infected wounds, ultimately promoting diabetic wound repair.

### 2.16 Further Analyze the Mechanism by Which CF‐CPGaMPN Hydrogel Promotes the Healing of Diabetic Infection Wounds

**FIGURE 10:**
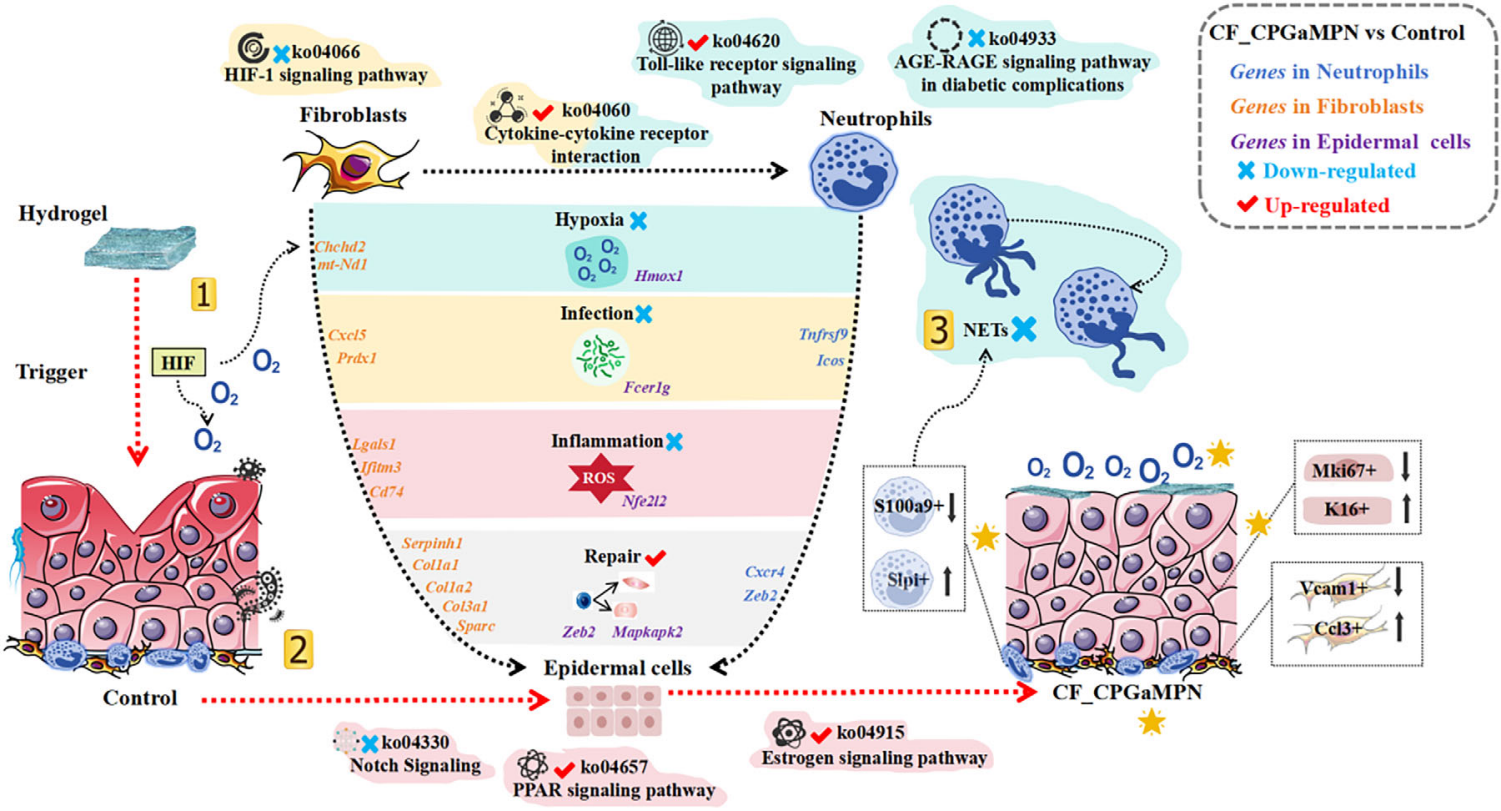
The mechanism diagram of CF‐CPGaMPN hydrogel promoting the healing of diabetic infected wounds.

This study explored the mechanism of CF‐CPGaMPN hydrogel in repairing diabetic infected wounds (Figure 10). This process involves complex regulation of multiple cell types, signalling pathways and changes in gene expression. The research mainly focuses on three aspects: alleviating the hypoxic environment of wounds, eliminating bacterial infections, and inhibiting Neutrophil NETs.

Diabetic infection wounds are often accompanied by hypoxic environments. Therefore, alleviating tissue hypoxia can promote the healing of diabetic infection wounds. First, after CF‐CPGaMPN hydrogel comes into contact with the wound, it can release oxygen, thereby causing the down‐regulation of the HIF‐1 signalling pathway (ko04066) related to the hypoxic environment in Fibroblasts, which is conducive to the repair and regeneration of Epithelial tissues. Moreover, cellular metabolism shifted from glycolysis to mitochondrial oxidative metabolism, thereby promoting the significant expression of genes Chchd2, mt‐Nd1 and Hmox1. This is crucial for the energy metabolism adjustment of cells around the wound and the promotion of wound angiogenesis. Second, after hydrogel treatment, the genes Cxcl5, Prdx1, Tnfrsf9, and Icos that resist bacterial infection in Fibroblasts and Neutrophils were significantly expressed. This leads to the upregulation of Cytokine‐cytokine receptor interaction (ko04060), a signalling pathway that activates immune cells by stimulating Fibroblasts and Neutrophils to secrete cytokines such as IL‐1β and TNF‐α. It enhances the immune response of cells around the wound to bacteria. Meanwhile, these factors can also regulate the activity and function of immune cells and improve the body's antibacterial ability. In addition, the Toll‐like receptor signalling pathway (ko04620) in Neutrophils is also significantly upregulated, which can induce the production of antimicrobial peptides, such as β ‐defensins, etc. These antimicrobial peptides can directly kill bacteria, enhance the body's antibacterial ability, and protect wounds from bacterial infection. Meanwhile, the AGE‐RAGE signalling pathway (ko04933) was downregulated in Neutrophils. The inhibition of this pathway helps to reduce the inflammatory response of cells around the wound, lower oxidative stress, protect cell function, promote angiogenesis and extracellular matrix remodeling. This is conducive to the repair of diabetic wounds. In addition, after treatment with CF‐CPGaMPN hydrogel, the anti‐inflammatory related genes Lgals1, Ifitm3, Cd74 and Nfe2l2 were significantly expressed in the three types of cells. Genes related to wound repair, such as Serpinh1, Col1a1, Col1a2, Col3a1, Col5a2, Sparc, Mapkapk2, Cxcr4 and Zeb2, were also significantly expressed in the three types of cells. Meanwhile, the down‐regulation of Notch Signaling (ko04330) in Epidermal cells helps to alleviate inflammation, promote angiogenesis, enhance cell proliferation and migration, etc., thereby accelerating the repair of diabetic infected wounds. The upregulation of the PPAR signaling pathway (ko04657) and Estrogen signaling pathway (ko04915) in Epidermal cells also contributes to the accelerated healing of diabetic wounds. To further verify the downregulation of the key signaling pathways observed, we also conducted an immunofluorescence (IF) experiment to detect the expression of HIF‐1α and Notch1. The results (Figure S12) clearly show that compared to the control group, their expression was significantly reduced in the CF‐CPGaMPN hydrogel group, directly confirming the conclusion of pathway downregulation.

The pivotal role of Neutrophil extracellular traps (NETs) in wound repair should not be overlooked. The CF‐CPGaMPN hydrogel effectively ameliorates the diabetic wound microenvironment by downregulating S100a9+ Neutrophils and upregulating Slpi+ Neutrophils, thereby suppressing NETs formation. This mechanism significantly reduces inflammation, oxidative stress, and cellular damage while promoting angiogenesis and ECM remodeling, ultimately accelerating the wound healing process. Furthermore, the CF‐CPGaMPN hydrogel improved the diabetic wound microenvironment by upregulating Ccl3+ Fibroblasts and K16+ Epidermal cells while downregulating Vcam1+ Fibroblasts and Mki67+ Epidermal cells, thereby promoting diabetic wound healing.

All in all, CF‐CPGaMPN hydrogel can alleviate wound hypoxia, eliminate bacterial infection and inhibit NETs in the microenvironment of diabetic infected wounds. The specific process involves the regulation of multiple cell phenotypes, activation or inhibition of signalling pathways and changes in gene expression. These complex physiological processes interact with each other and ultimately jointly promote the healing of diabetic infection wounds  .

## 3 Conclusion

In this study, we have developed a glucose‐triggered on‐demand drug delivery smart hydrogel system based on boronate ester bonds, named CF‐CPGaMPN hydrogel. The CF‐CPGaMPN hydrogel not only effectively inactivates various microorganisms and continuously releases oxygen, but also features boronate ester bonds that can be cleaved in a high‐glucose environment, enabling glucose‐triggered on‐demand drug release for diabetic infected wound healing. Additionally, the hydrogel exhibits excellent hemostatic and self‐healing properties, all of which are crucial for the healing of diabetic infected wounds. Single‐cell sequencing revealed that the heterogeneity of Neutrophils, Fibroblasts, and Epidermal cells, observing that CF‐CPGaMPN hydrogel played a significant role in mitigating the dysfunctional Neutrophils, Fibroblasts, and Epidermal cells in the diabetic microenvironment. Furthermore, CF‐CPGaMPN hydrogel accelerated diabetic wound healing by inhibiting the formation of NETs and alleviating cellular hypoxia. In summary, CF‐CPGaMPN hydrogel not only provides a promising reactive drug release strategy for dressings to promote healing of diabetic infected wounds, which has great potential for application in the treatment of diabetic infected wounds, but also helps to elucidate the key molecular mechanisms and identify new therapeutic targets for different cellular subpopulations so as to rationalize the design of smart hydrogels.

## 4 Experimental Section

### 4.1 Methods and Reagents

All reagents were obtained from commercial suppliers and were used without further purification. Gallium chloride anhydrous, 2‐FPBA, carboxymethyl chitosan (CMCS), and CaCl_2_ were purchased from Aladdin (USA). Tannic acid (TA), Catalase, Diethyldithiocarbamate (DEDTC), and PBS powder were purchased from Macklin (China). NH_4_OH, H_2_O_2_, and PVP were purchased from Sigma (USA). Glutaraldehyde (50% aqueous solution), streptozotocin (STZ; ≥ 98.0%) were purchased from Adamas‐beta (China). Foetal bovine serum (FBS) and dulbecco's Modified Eagle's Medium (DMEM) were purchased from Gibco (USA). Luria‐Bertani broth (LB broth) was from Hopebio (China). Live and dead bacterial staining kits were provided by Molecular Probes (USA). Cell Counting Kit‐8 (CCK‐8), triton x‐100 and calcein‐AM/propidium iodide (PI) were purchased from Beyotime Biotechnology (China). 4% paraformaldehyde was provided by Solarbio (China). Other organic solvents were purchased from Beijing Chemical Factory (China).

### 4.2 Synthesis and Physicochemical Characterization of GaMPN Nanoparticles

GaMPN nanoparticles were synthesised using a one‐step assembly method. Briefly, the pH of TA aqueous solution (600 µL, 2 mM) was first adjusted to 8–9 with NaOH solution (1 M), and then DEDTC (600 µL, 2 mM) was added. Next, GaCl_3_ aqueous solution (600 µL, 2 mM) was added dropwise to the reaction vessel to start the assembly of GaMPN nanoparticles. The product was collected by centrifugation at 7000 rpm to remove minute aggregates, free TA, DEDTC and metal ions.

The attenuated total reflection FT‐IR spectra of TA, DEDTC and GaMPN were measured on an infrared spectrometer (Bruker VERTEX 80V, Germany), where the scanning range was set from 500 to 4000 cm^−1^. The ^1^H nuclear magnetic resonance (^1^H NMR) (400 MHz, Deuterium Oxide) spectra of TA, DEDTC and GaMPN were tested using a spectrometer (Zhongke Oxford 400MHz Spectrometer, China) to verify the successful synthesis of the material. The micromorphology of GaMPN nanoparticles was observed by SEM (Thermo Fisher Scientific Apreo 2C, USA). Prior to examination, all samples were freeze‐dried and sprayed with a thin layer of gold. The diameters of the GaMPN nanoparticles were measured using Nano Measure (n = 100). The morphology and elemental composition of the GaMPN nanoparticles were further examined using transmission electron microscopy (TEM, Thermo Fisher Scientific Talos‐F200S) coupled with energy dispersive X‐ray spectroscopy (EDS). The surface morphology of GaMPN was measured using an atomic force microscope (AFM, Bruker Dimension FastScan AFM, Germany). XRD (Rigaku Ultima IV, Japan) was used to analyze the phase composition of the GAMPN samples. X‐ray powder diffraction samples were prepared by placing a drop of GaMPN suspension or Ga^3+^ solution on glass and dried overnight. The surface chemical groups and elemental composition of GaMPN nanoparticles were determined using X‐ray photoelectron spectroscopy (XPS, Thermo Fisher Scientific K‐ALPHA, USA).

### 4.3 Synthesis and Physicochemical Characterization of PVP@CaO_2_ Nanoparticles

PVP (0.35 g) and CaCl_2_ (0.1 g, 0.68 mmol) were combined and dissolved in 15 mL of ethanol under ultrasonication. Subsequently, 1 mL of NH_4_OH (0.8 M) was introduced under continuous stirring. Following this, 0.2 mL of H_2_O_2_ (1 M) solution was infused at a steady rate of 0.05 mL·min^−^^1^, resulting in a light blue milky suspension. The resulting product was recovered by centrifugation at 11 000 rpm, washed three times with ethanol, and ultimately redispersed in 5 mL of ethanol to yield PVP@CaO_2_ nanoparticles.

The attenuated total reflection FT‐IR spectra of PVP@CaO_2_ nanoparticles were measured on an infrared spectrometer (Bruker VERTEX 80V, Germany), where the scanning range was set from 500 cm^−1^ to 4000 cm^−1^. The micromorphology of PVP@CaO_2_ nanoparticles was observed by SEM (Thermo Fisher Scientific Apreo 2C, USA). The diameters of the PVP@CaO_2_ nanoparticles were measured using Nano Measure (n = 100). Prior to examination, all samples were freeze‐dried and sprayed with a thin layer of gold. The morphology of PVP@CaO_2_ nanoparticles was further observed by TEM (Thermo Fisher Scientific Talos‐F200S, USA) and the elemental composition was observed by EDS. The oxygen concentration measurements were obtained by dissolved oxygen meter (AR8010+, China). CaO_2_、CaO_2_+Catalase and PVP@CaO_2_ +Catalase were added into the water. Subsequently, the probe was inserted into water and liquid paraffin was dripped into the system. The surface chemical groups and elemental composition of PVP@CaO_2_ nanoparticles were determined using XPS (Thermo Fisher Scientific K‐ALPHA, USA).

### 4.4 Synthesis and Physicochemical Characterization of CF‐CPGaMPN Hydrogels

Initially, 2‐FPBA powder was dispersed in 0.01 M PBS (pH = 7.4) under ultrasonication at room temperature to form a 2.8 wt% solution. Subsequently, a 5 wt% CMCS solution was obtained by dissolving CMCS powder in deionized water under continuous stirring at room temperature. Then, the CMCS and 2‐FPBA solutions were mixed at a volume ratio of 8:1 at room temperature, followed by doping with 1 wt% of GaMPN nanoparticles, 2 wt% of CP nanoparticles, and 2 wt% of catalase, and then the mixtures were rapidly vortexed to obtain CF‐CPGaMPN hydrogels. Here, C represents CMCS, F represents 2‐FPBA, and CP represents PVP@CaO_2_ nanoparticles mixed with catalase.

The attenuated total reflection FT‐IR spectra of CMCS, 2‐FPBA and CF were measured on an infrared spectrometer (Bruker VERTEX 80V, Germany) with the scanning range set from 500 to 4000 cm^−1^. The macromorphology of CF hydrogel was observed by photographing. The micromorphology of CF hydrogels was observed by SEM (Thermo Fisher Scientific Apreo 2C, USA). Prior to examination, all samples were freeze‐dried and sprayed with a thin layer of gold. The elemental composition of CF hydrogels was determined by EDS. The measurement of oxygen concentration is carried out using a dissolved oxygen meter (AR8010+, China). CF hydrogel and CF‐CPGaMPN hydrogel are added to the water. Subsequently, the probe is inserted into the water, and liquid paraffin is dropped into the system.

### 4.5 Self‐Healing and Injection Tests of CF Hydrogels

All rheological tests were performed on a rheometer (Waters Corporation, China). The values in the critical strain region were recorded using the strain amplitude scanning method (γ from 0.01% to 1000%). The self‐healing behavior of the CF hydrogels was further evaluated through alternating strain sweep tests conducted at a constant angular frequency of 10 rad s^−^^1^. The applied oscillatory strain was alternated between a low amplitude (γ = 1.0%, 100 s intervals) and a high amplitude (γ = 1000%, 100 s intervals), with the cycle repeated four times.

In the macroscopic self‐healing experiment, two pieces of hydrogels were dyed with different colored dyes and cut in half. The cuts of the hydrogels were then allowed to contact each other for 5 min, and the self‐healing process of the hydrogels was photographed and recorded. To test the injectability of the hydrogel, we filled a 1 ml syringe with the hydrogel to form a specific shape.

### 4.6 Glucose‐Responsive Release In Vitro

The cross‐linked structure of the boronic ester bond is sensitive to glucose, subsequently enhancing the degradation of the hydrogel and promoting the release of GaMPN nanoparticles. In order to study the glucose‐responsive release characteristics of GaMPN nanoparticles, 1 milliliter of CF‐CPGaMPN hydrogel was added to 100 milliliters of PBS containing 0 mmol, 5.6 mmol, 10 mmol and 16.7 mmol of glucose, and it was placed in a shaker running at a speed of 100 revolutions per minute. At various time points, 100 µL of the supernatant was extracted, and the absorbance at 330 nm was recorded using a UV–visible spectrophotometer to quantify the amount of GaMPN nanoparticles

### 4.7 The Degradation Properties of All Hydrogels

The degradability of CF hydrogel, CF hydrogel loaded with PVP@CaO_2_ and Catalase (CF‐CP), CF hydrogel loaded with PVP@CaO_2_,Catalase and Ga^3+^ (CF‐CPGa) and CF hydrogel loaded with PVP@CaO_2_,Catalase and GaMPN (CF‐CPGaMPN) was tested via the gravimetric method. Each group of hydrogel was placed in a 50 mL centrifuge tube and added with 40 mL PBS solution (0.02 M) as degradation solution. The mixture was incubated at 37°C. At predetermined time intervals, the degradation solution was removed, and the remaining hydrogel was weighed. All samples were prepared in triplicate. Calculate according to Equation (1):

(1) $$\mathrm{Degradation}=\left(W_{d}-W_{s}\right)/W_{d}\times 100\%$$

W_s_: weight of hydrogel at different times; W_d_: initial weight of hydrogel.

### 4.8 Swelling Behavior of All Hydrogels

The swelling ratio was determined via the gravimetric method. Each group of freeze‐dried hydrogel was weighed and placed in a 50 mL centrifuge tube containing 37°C PBS solution. Swollen hydrogels were removed at predetermined time intervals and weighed quickly after the removal of water from the surface. All samples were obtained in triplicate. Calculations according to Equation (2):

(2) $$\mathrm{Swelling}=\left(W_{s}-W_{d}\right)/W_{d}\times 100\%$$

W_s_: weight of hydrogel at different times; W_d_: initial weight of freeze‐dried hydrogel.

### 4.9 Cytocompatibility of CF‐CPGaMPN Hydrogels

L929 cells (ATCC, USA) were propagated in α‐MEM complete medium, supplemented with 10% FBS and 1% penicillin/streptomycin. Following dissemination into 96‐well plates at 5000 cells per well, the cultures were administered 4 µL of individual hydrogels: CF, CF‐CP, CF‐CPGa, and CF‐CPGaMPN. A control group without any hydrogel was also included. Cell viability was evaluated using a CCK‐8 assay following the supplier's protocol. In brief, after washing the cells three times with PBS at the designated time point (1 day), 110 µL of medium with 10% (v/v) CCK‐8 solution (without FBS) was added to each well and incubated for 60 min. Following transfer of 100 µL of medium from each well to a fresh 96‐well plate, absorbance was read at 450 nm with a microplate reader to determine cell viability and proliferation.

To visualize cell growth, L929 cells cultured for 1, 2, and 3 days were stained with a live/dead staining solution (Calcein AM/PI) for 30 min at 37°C. Fluorescence images were acquired using a fluorescence microscope (DP74, OLYMPUS, China).

In order to observe the adhesion of L929 cells to the bottom of the well plate and the cell morphology after co‐culture with different hydrogel sets for 72 h, the cytoskeleton of L929 cells was stained with rhodamine and DAPI at 37°C. The cytoskeleton and cell morphology were observed with a fluorescence microscope (DP74, OLYMPUS, China).

### 4.10 Haemocompatibility of CF‐CPGaMPN

The haemocompatibility of CF‐CPGaMPN was evaluated through a haemolysis assay. Fresh mouse blood (500 µL) was collected and diluted with 5 mL of PBS, which was ten times the volume of the blood sample, followed by centrifugation at 1000 rpm for 5 min. After discarding the supernatant, the pellet was resuspended in 10 mL of PBS and centrifuged again under the same conditions. This washing procedure was repeated once more. Finally, the purified red blood cells were resuspended in 10 milliliters of PBS. A 200 µL volume of erythrocyte suspension was mixed with 800 µL of 2% Triton X‐100 for the positive control, and with 800 µL of PBS for the negative control. For the hydrogel treatment groups (CF, CF‐CP, CF‐CPGa, CF‐CPGaMPN), 200 µL of erythrocyte suspension was incubated with 800 µL of PBS in the presence of each material. Following a 2‐h incubation at 37°C, the samples were centrifuged (1000 rpm, 5 min). From each, 100 µL of supernatant was collected and transferred to a 96‐well plate for absorbance measurement at 577 nm on a microplate reader. Haemolysis rate calculated according to Equation (3):

(3) $$\mathrm{Hemolysis}\;\mathrm{rate}=\left(A-B\right)/\left(C-B\right)\times 100\%$$

A: Absorbance of the experimental group, B: Absorbance of the negative control group, C: Absorbance of the positive control group.

### 4.11 In Vitro Haemostatic Performance Test

CF, CF‐CP, CF‐CPGa, CF‐CPGaMPN hydrogels were pre‐warmed in deionized water at 37°C in advance and placed in centrifuge tubes. To each hydrogel group and the negative control plate, a mixture of 0.1 mL mouse whole blood and 0.01 mL sodium citrate solution (38 mg mL^−^^1^) was subsequently applied, ensuring complete coverage of the surface. Coagulation was initiated by adding 10 µL of calcium chloride solution (concentration 0.2 M), and the temperature of the procedure was maintained at 37°C. After initiating blood coagulation at 37°C for 5 min, 25 ml deionized water was added to the culture dish. The supernatant was collected after the incubation for 10 min, the supernatant obtained was transferred to a new centrifuge tube, and the absorption of the solution at 540 nm was recorded by an enzyme marker. The coagulation index (BCI) was calculated according to Equation (4):

(4) $$\mathrm{BCI}\left(\%\right)=OD_{\mathrm{sample}}/OD_{\mathrm{reference}\;\mathrm{value}}\times 100\%$$

OD_sample_: Absorbance of the solution from the samples at 540 nm, OD_reference value_: Absorbance of the solution from the negative control at 540 nm.

### 4.12 In Vivo Haemostatic Effect Evaluation

A mouse liver injury haemorrhage model was used to investigate the in vivo haemostatic effect of hydrogel. First, the mice were anaesthetized with isoflurane, and after anesthesia, the mice were fixed on a cork board and their livers were exposed through the abdominal incision. The cork board was then tilted at about 30° and a 20 G needle was used to cause the liver to bleed, and the hydrogel was immediately applied to the bleeding site, with a filter paper underneath the liver (the filter paper was replaced every 15 s).

### 4.13 Bacterial Culture and the CFU Experiment

*E. coli* (BNCC 133264), *S. aureus* (BNCC 186335) and *MRSA* (ATCC 43300) were grown in LB broth at 37°C. A bacterial suspension containing three strains (with a concentration of 1.0 × 10^6^ CFU per milliliter) was introduced into a 24‐well plate (1 milliliter per well), with each well pre‐installed with a circular glass petri dish. After culturing for 48 h to form a biofilm, 100 microliters of a type of hydrogel (CF, CF‐CP, CF‐CPGa or CF‐CPGaMPN) was added to each well. Then, the samples were incubated at 37°C for 48 h. Afterward, the glass culture dishes were transferred to 1 milliliter of sterile PBS and subjected to ultrasonic treatment to separate the attached bacteria. The detached bacterial suspensions were then serially diluted up to 1000‐fold. Aliquots of 50 µL from the dilutions were plated onto agar plates and incubated at 37°C for 24 h. Bacterial colonies were enumerated and photographed for subsequent analysis.

### 4.14 Scanning Electron Microscopy

The bacterial culture procedure was the same as for the CFU experiments, after removing the adherent bacteria from the surface of the sections by ultrasound, the resulting bacterial suspension was dropped on the silica sheets, and 2.5% glutaraldehyde was added to immobilize the bacterial morphology for 12 h. Then it was further dehydrated by 30, 40, 50, 60, 70, 80, 90, 95 and 100% ethanol solutions. Following dehydration, the specimens were subjected to gold sputtering to ensure sufficient conductivity. Morphological analysis was subsequently carried out using a scanning electron microscope.

### 4.15 Live‐Dead Staining

Each well of a 24‐well plate, which contained a circular glass climbing section, was inoculated with 1 mL of an *E. coli*, *S. aureus* and *MRSA* suspension at a concentration of 1.0 × 10^6^ CFU mL^−^^1^. The plates were subsequently incubated with the different hydrogel groups for 48 h at 37°C. The bacteria on the glass climbing sections were then stained with bacterial live‐dead stain (SYTO 9 and propidium iodide (PI)) and kept in the dark for 20 min, followed by washing the glass climbing sections three times with PBS. Confocal laser scanning microscopy was employed to acquire 3D images of the biofilms, which then underwent processing and analysis with Image J software for quantification.

### 4.16 Anti‐Biofilm Evaluation

To test the ability of each group of hydrogels to destroy the biofilm, 1 milliliter of the suspension of live Staphylococcus aureus and Escherichia coli (with a concentration of 1 × 10^6^ CFU mL^−1^) was added to a 24‐well plate. Then, the plate was incubated at 37°C for 48 h. Subsequently, 100 microliters of CF hydrogel, CF‐CP hydrogel, CF‐CPGa hydrogel and CF‐CPGaMPN hydrogel were added to the wells, and the plate was incubated at 37°C for another 48 h. After three PBS washes of the biofilm, it was fixed with 4% paraformaldehyde for 10 min, and then stained with 0.1% crystal violet staining solution for 30 min. Finally, the biofilm images were captured after being washed with PBS and dried. To quantitatively detect the content of the biofilm, the dye was dissolved in 33% acetic acid, and the absorbance of the enzyme‐labeled solution at 590 nanometers was measured.

### 4.17 Diabetic Mouse Model and In Vivo Wound Healing Assay

For wound healing experiments, 45 male ICR mice weighing 25–35 g and 5–6 weeks old were selected to evaluate the wound healing efficiency of each hydrogel group. All mice were randomly divided into five groups: control, CF, CF‐CP, CF‐CPGa and CF‐CPGaMPN. All procedures involved in the animal experiments were approved by the Ethics Committee for Medical Laboratory Animals of West China College of Stomatology, Sichuan University (Chengdu, China), with the ethical approval number (WCHSIRB‐AT‐2025‐381). Mice were injected intraperitoneally with 100 µL of streptozotocin (STZ) (50 mg kg^−1^). Blood glucose levels in venous blood of non‐fasting animals were measured using a glucometer (Yuwell, China) for 5 consecutive days after 12 days. When blood glucose levels consistently exceeded 16.7 mM L^−1^, mice were assessed and considered diabetic. These diabetic mice were used to establish a diabetic wound model. To create a wound infection model in mice, the animals were first anesthetized using isoflurane. The dorsal fur of each mouse was then closely shaved, and any residual hair was eliminated with a depilatory agent. Following skin preparation, a circular full‐thickness excisional wound, measuring about 7 mm across, was created on the back. Each wound was subsequently inoculated with 50 µL of a *S. aureus* bacterial suspension containing 1 × 10^6^ CFU per milliliter. After inoculation, a hydrogel dressing was applied to cover the wound site. The hydrogel dressings were replaced every 72 h. Wound images were captured on days 0, 2, 4, 7, 10, and 14 post‐operation using a digital camera. These photographs were subsequently processed and measured using ImageJ software. The percentage of wound closure for each experimental group was determined based on Equation (5):

(5) $$\mathrm{Healing}\;\mathrm{rate}\left(\%\right)=\left(S_{0}-S_{t}\right)/S_{0}\times 100\%$$

S_0_: initial wound area, S_t_: remaining wound area at each time point.

In this study, in addition to establishing an infection model using *S. aureus*, we also conducted a parallel animal experiment by inoculating wound sites with 50 µL of an *MRSA* bacterial suspension containing 1 × 10^8^ CFU mL^−1^ in diabetic mouse wound healing models. A total of 36 male ICR mice, aged 5–6 weeks and weighing 25–35 g, were randomly divided into six groups: Control, CF, CF‐CP, CF‐CPGa, CF‐CPGaMPN, and Biatain Alginate Ag, to systematically evaluate the effects of different treatments on the healing of diabetic wounds infected with *MRSA*.

### 4.18 Histopathological Studies

On days 7 and 14 post‐injury, mice were euthanized and skin samples from the wound area were harvested. After fixation in 4% paraformaldehyde, the tissues were paraffin‐embedded and sectioned at 4 µm. These sections were processed for both standard H&E staining and Masson's trichrome staining (MTS). Angiogenesis and inflammatory responses were further assessed via immunofluorescence staining targeting CD31 and Il‐1β. To assess the acute toxicity of the materials in mice, major organs were collected and stained with H&E for histological analysis, including the heart, liver, spleen, lungs and kidneys. Blood samples were also collected from the experimental mice, and their liver and kidney functions were tested. All images were performed using an inverted fluorescence microscope (TE2000, Nikon, Japan).

### 4.19 Single‐Cell RNA Sequencing

The wound tissue was cut from the surrounding edges and healthy tissues, and then minced using scissors. It was subsequently subjected to digestion treatment. After stopping the digestion process with the addition of fetal bovine serum, the living cells were isolated from the cell suspension. Cell processing steps including washing, counting, and concentration—for both high and low cell yields (above or below 100 000 cells)—followed the recommendations outlined in the 10× Genomics Cell Preparation Guide. ScRNA‐seq was performed using the Chromium system from 10× Genomics. Cell suspensions were loaded onto microfluidic chips (3′ v2 chemistry) and barcoded with the 10× Chromium Controller. Reverse transcription and construction of sequencing libraries were carried out using the Chromium Single Cell 3′ v2 reagent kit in accordance with the manufacturer's protocols. The resulting libraries were sequenced on Illumina platforms (either HiSeq 2000 or NovaSeq, depending on the project) following standard Illumina procedures.

### 4.20 scRNA‐Seq Data Processing

The analysis process of 10× Genomics single‐cell transcriptome sequencing information is mainly divided into three parts: sequencing data quality control, Cell Ranger for gene expression quantification, Seurat for downstream cell clustering, and differential expression gene analysis. Among them, sequencing data quality control includes filtering the sequences obtained from sequencing, evaluating the quality of sequencing data, and calculating the distribution of sequence length; The Cell Ranger analysis process is a professional analysis software developed for 10× Genomics single‐cell transcriptome sequencing, including data splitting, cell count statistics, gene expression quantification, clustering, and differential analysis. Cell Ranger gene expression quantification and Seurat cell clustering mainly use cell barcode and UMI information to analyze gene expression levels in single cells. Due to the fact that Cell Ranger did not filter out low‐quality data during subsequent gene expression analysis, a third‐party analysis tool Seurat was used to filter out low‐quality data from the expression matrix and perform subsequent expression analysis. KEGG enrichment analysis was performed on the differentially expressed genes obtained from Seurat analysis. Subsequently, through immunofluorescence staining for Hif‐1α and Notch1, the downregulation of the pathway was further confirmed.

### 4.21 Cell–Cell Communication Analysis

We analyzed cell–cell communication by using the CellChat (version CellChat_1.5.0). We applied the “computeCommunProb” function to calculate the communication probability. We identified the signaling pathways responsible for cell‐cell communications by using the “computeCommunProbPathway” function.

### 4.22 Statistical Analysis

The results are expressed as the mean ± standard deviation. Analysis was calculated using one‐way ANOVA method with Tukey's post hoc test by GraphPad Prism Software. Significant differences were considered with *p* values below 0.05 and are indicated in the figures. *p < 0.05, **p < 0.01, ***p < 0.001, ****p < 0.0001. All experiments were repeated three times.

## Funding

This work was supported by the National Natural Science Foundation of China (82370951) and the Sichuan Science and Technology Program (2024YFHZ0008).

## Ethics Approval and Consent to Participate

This study and the included experimental procedures were approved by Ethics Committee for Medical Laboratory Animals of West China College of Stomatology, Sichuan University (Chengdu, China), with the ethical approval number (WCHSIRB‐AT‐2025‐381).

## Conflicts of Interest

The authors declare no conflict of interest.

## Supporting information




**Supporting File**: advs74085‐sup‐0001‐SuppMat.docx.

## Data Availability

The data that support the findings of this study are available from the corresponding author upon reasonable request.
