# Supplementary material for: A Glucose‐Responsive Intelligent Antibacterial and Oxygen‐Producing Hydrogel Promotes the Healing of Diabetic Wounds by Regulating Cellular Heterogeneity
Source: Adv Sci (Weinh). 2026 Jan 31;13(19):e17028. doi: 10.1002/advs.202517028 (PMC13045415; doi:10.1002/advs.202517028)
Supplement: Supplementary file 1 — Supporting File: advs74085‐sup‐0001‐SuppMat.docx. [file ADVS-13-e17028-s001.docx]

Supporting Information

A glucose-responsive intelligent antibacterial and oxygen-producing hydrogel promotes the healing of diabetic wounds by regulating cellular heterogeneity

Manxuan Liu^12^, Zhongcheng Li^12^, Qian Ren^12^, Ziqian Lu^12^, Yubing Zhang^12^, Yili Guo^12^, Ruomeng Li^12^, Die Hu^12,*^, and Linglin Zhang^12,*^


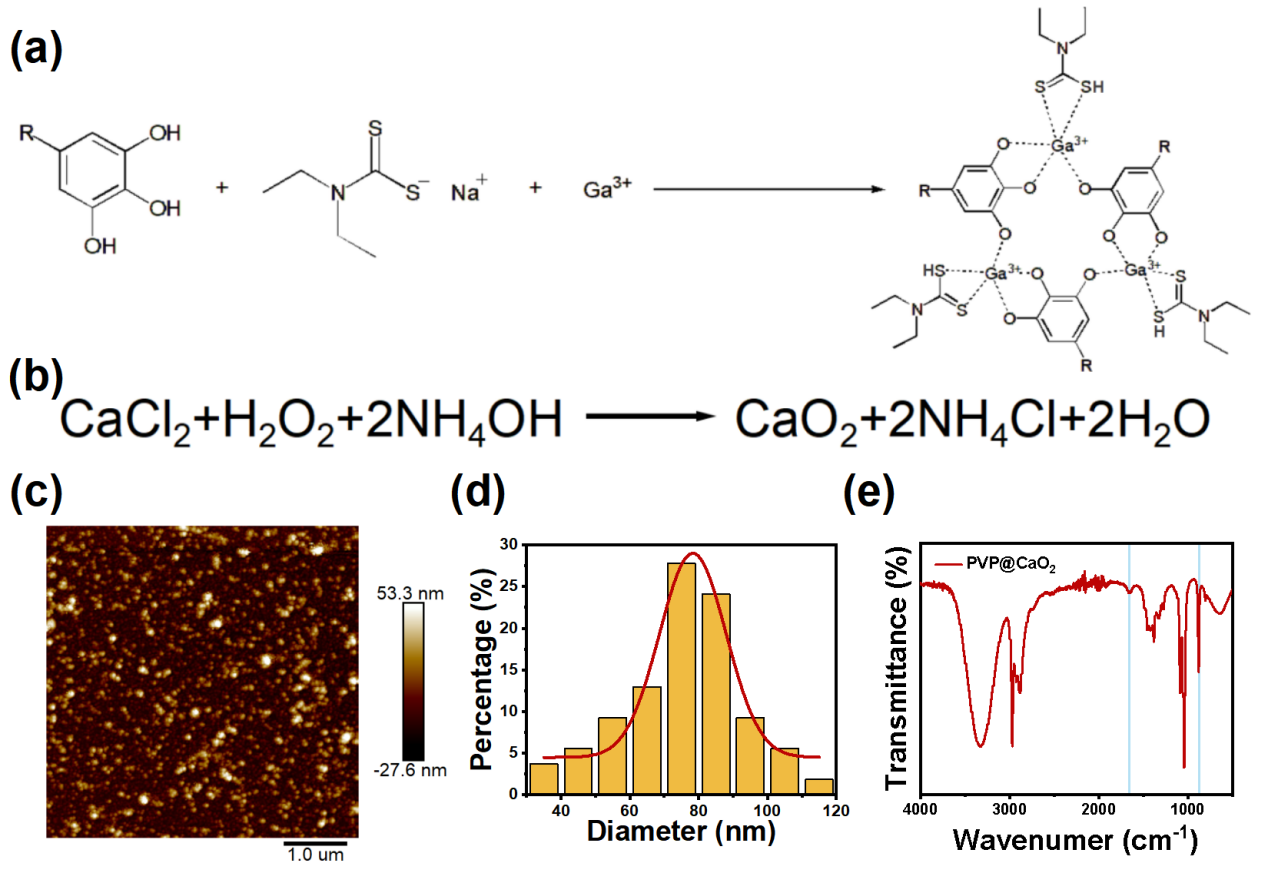


**Figure S1.** Characterisation of GaMPN nanoparticles and PVP@CaO_2_ nanoparticles. (a) Equation for GaMPN nanoparticles synthesis. (b) Equation for PVP@CaO_2_ nanoparticles synthesis. (c) AFM images of GaMPN nanoparticles. (d) Size distribution map of PVP@CaO_2_ nanoparticles. (e) FT-IR spectra of PVP@CaO_2_ nanoparticles.


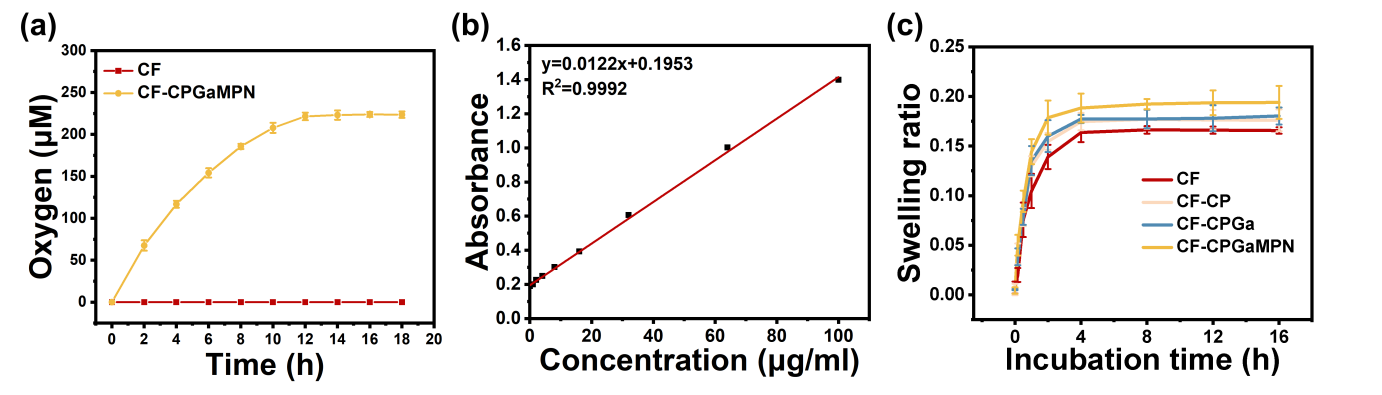


**Figure S2.** Characterisation of CF hydrogels. (a) Oxygen release from CF-CPGaMPN hydrogel. (b) The standard fitting curve for the concentrations of GaMPN nanoparticle to the relative absorbance. (c) The swelling curves of each group of CF hydrogels.


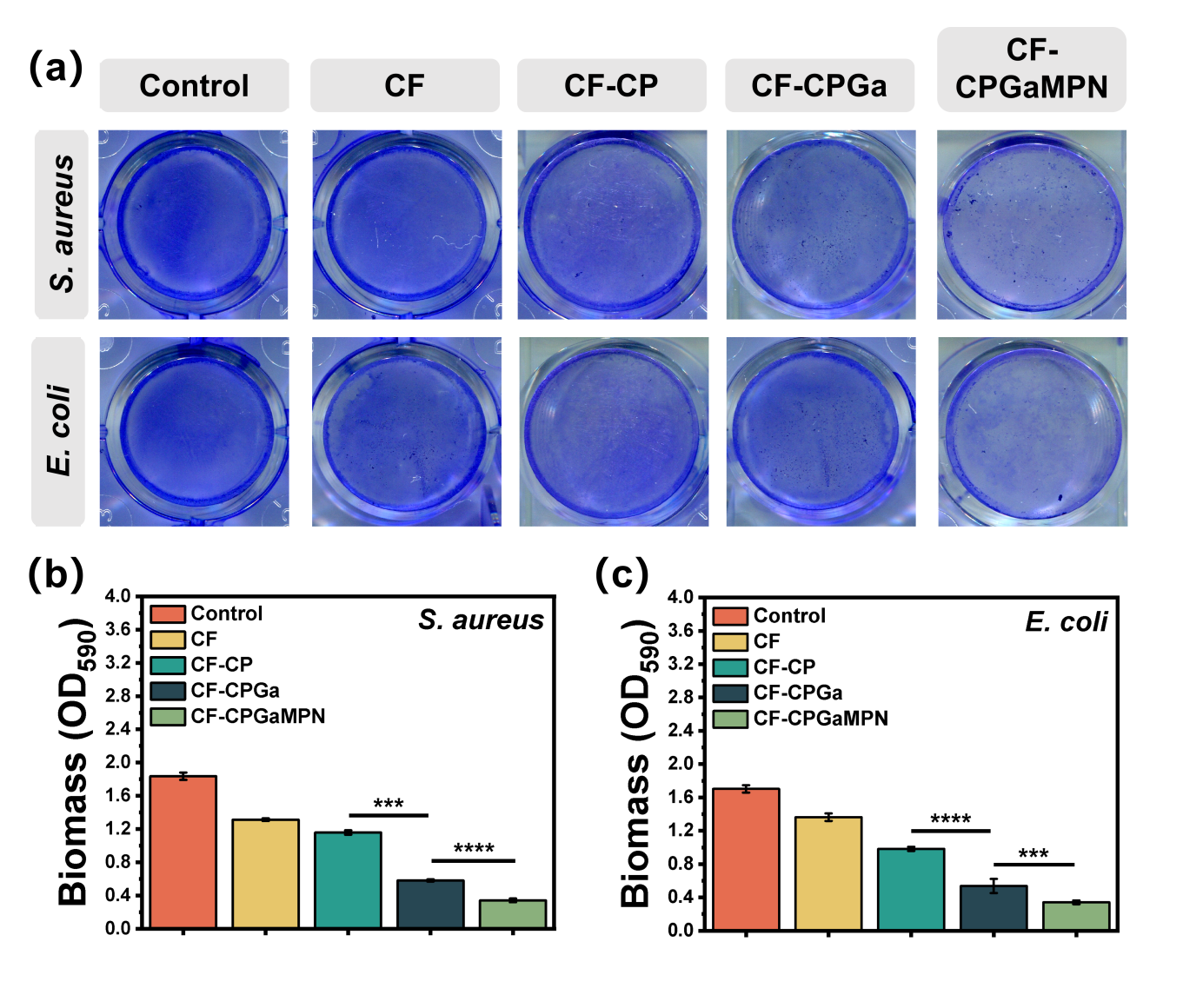


**Figure S3.** The effect of CF-CPGaMPN hydrogels on disrupting biofilms. a) Crystal violet staining of biofilms after various treatments. b, c) Biomass quantification corresponding to crystal violet staining.


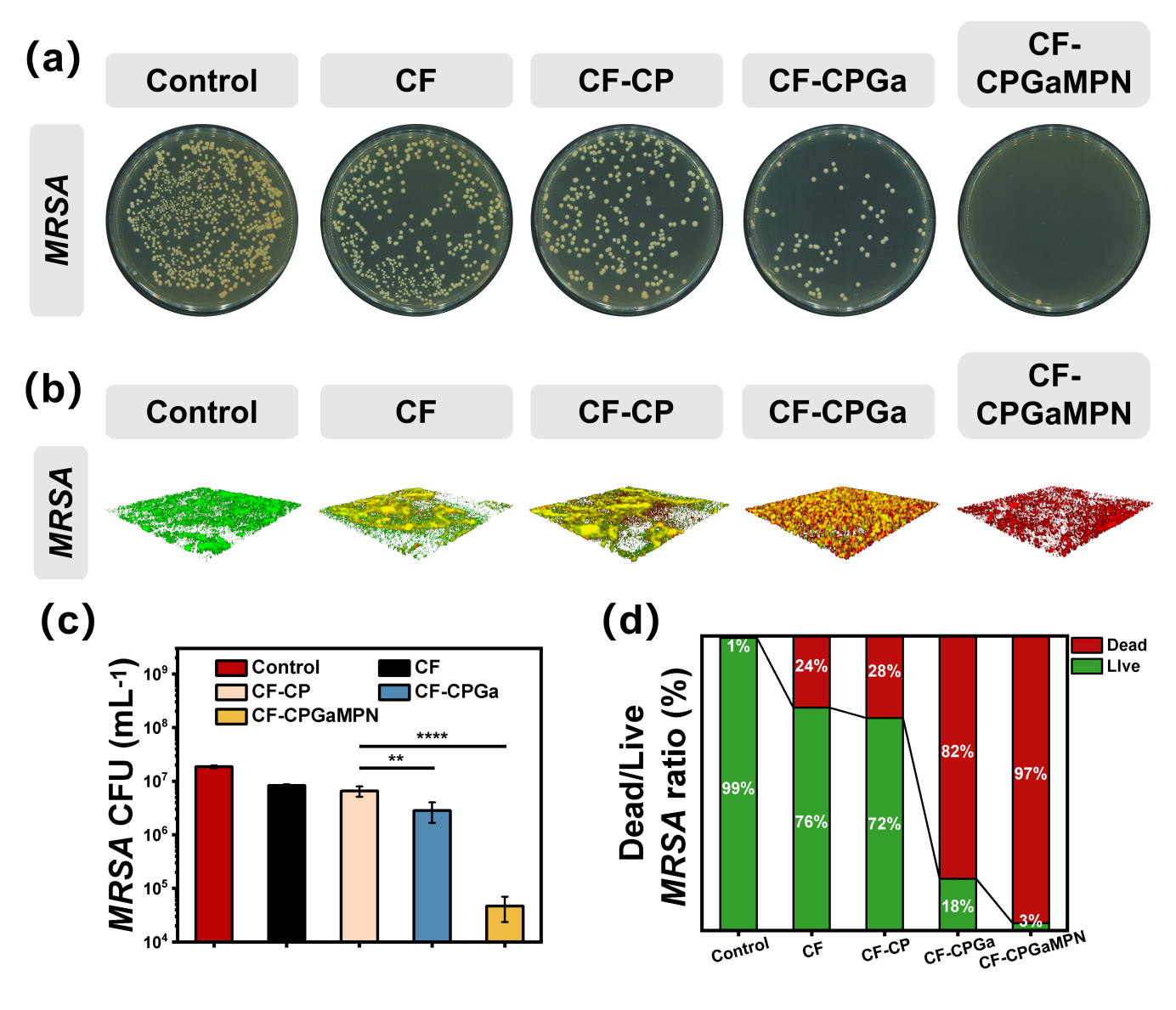


**Figure S4.** *In vitro* anti-*MRSA* bacterial efficacy of CF-CPGaMPN hydrogels. (a) CFU images of *MRSA* after different treatments. (b) Three-dimensional live/dead fluorescence staining of *MRSA* biofilms after treatment, with green and red fluorescence indicating live and dead bacteria, respectively. (c) Antimicrobial efficiency of each group of CF-CPGaMPN hydrogels against *MRSA*. (d) Corresponding proportions of live and dead *MRSA* in biofilms. *p < 0.05, **p < 0.01, ***p < 0.001, ****p < 0.0001, and n = 3.


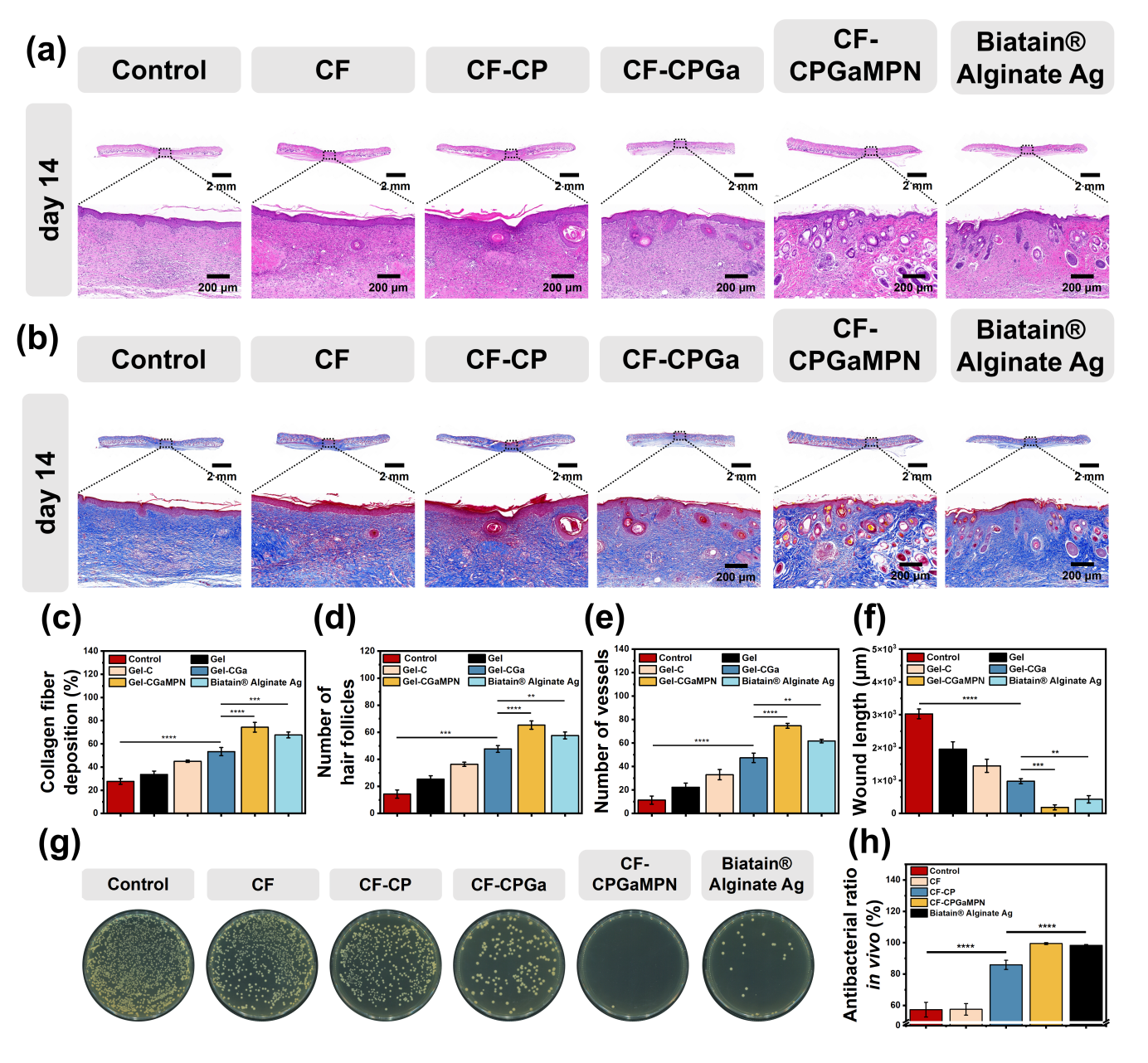


**Figure S5**. *In vivo* studies demonstrate the efficacy of CF-CPGaMPN hydrogels in enhancing the healing of *MRSA* infected skin tissue. (a) H&E staining of tissue sections from wound sites on day 14. (b) Masson’s trichrome staining of wound tissue sections obtained at day 14. (c) Quantitative data of the number of collagen fibers deposited. (d) Quantitative data of the number of hair follicles. (e) Quantitative data of the number of vessels. (f) Quantitative data of wound length. (g) *MRSA* CFU were visualized after two days of treatment. (h) Antibacterial ratio *in vivo*. *p < 0.05, **p < 0.01, ***p < 0.001, ****p < 0.0001, and n = 3.


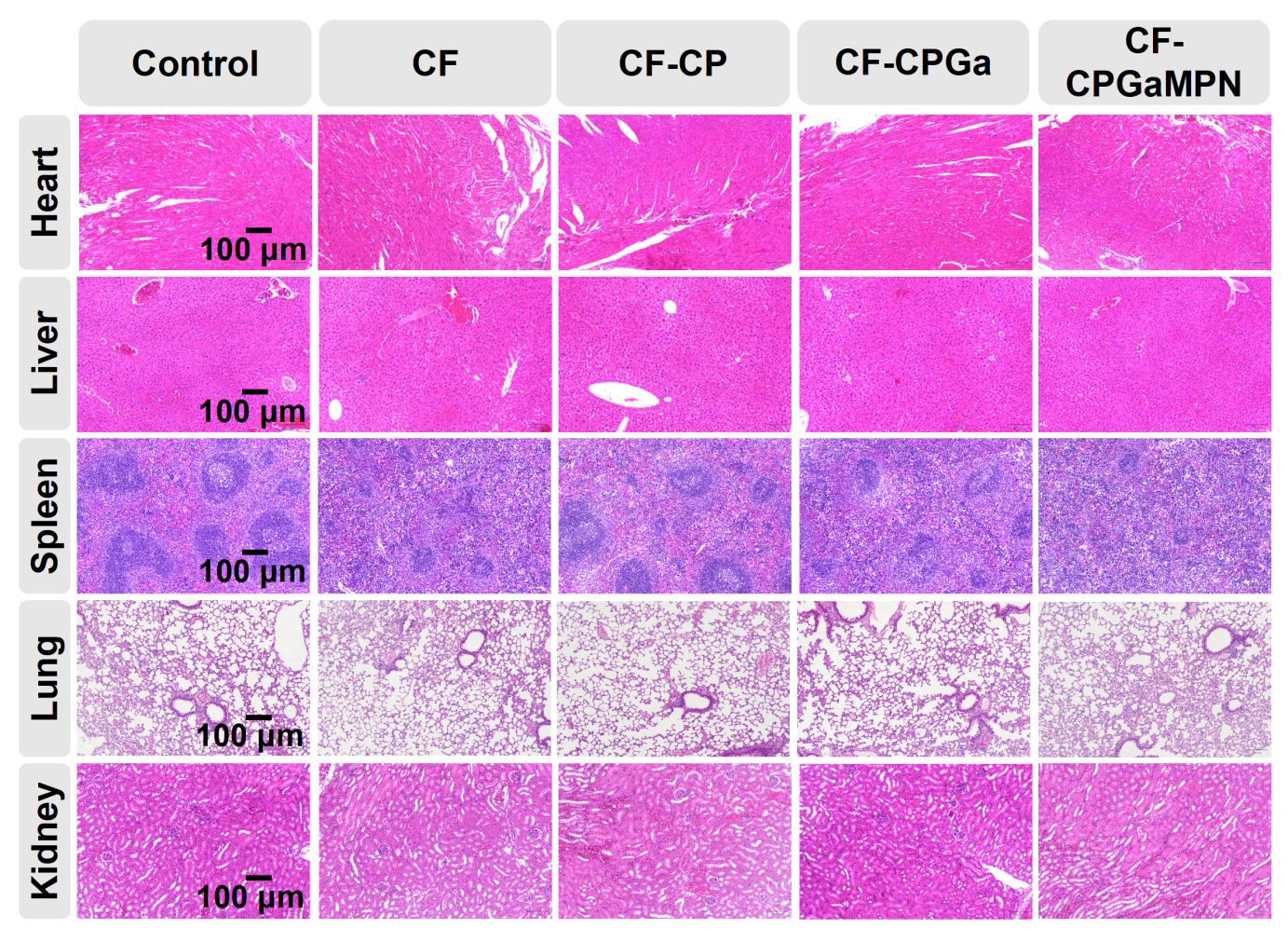


**Figure S6.** HE staining images of the major organs of the mice after two weeks of treatment with each group of hydrogels.


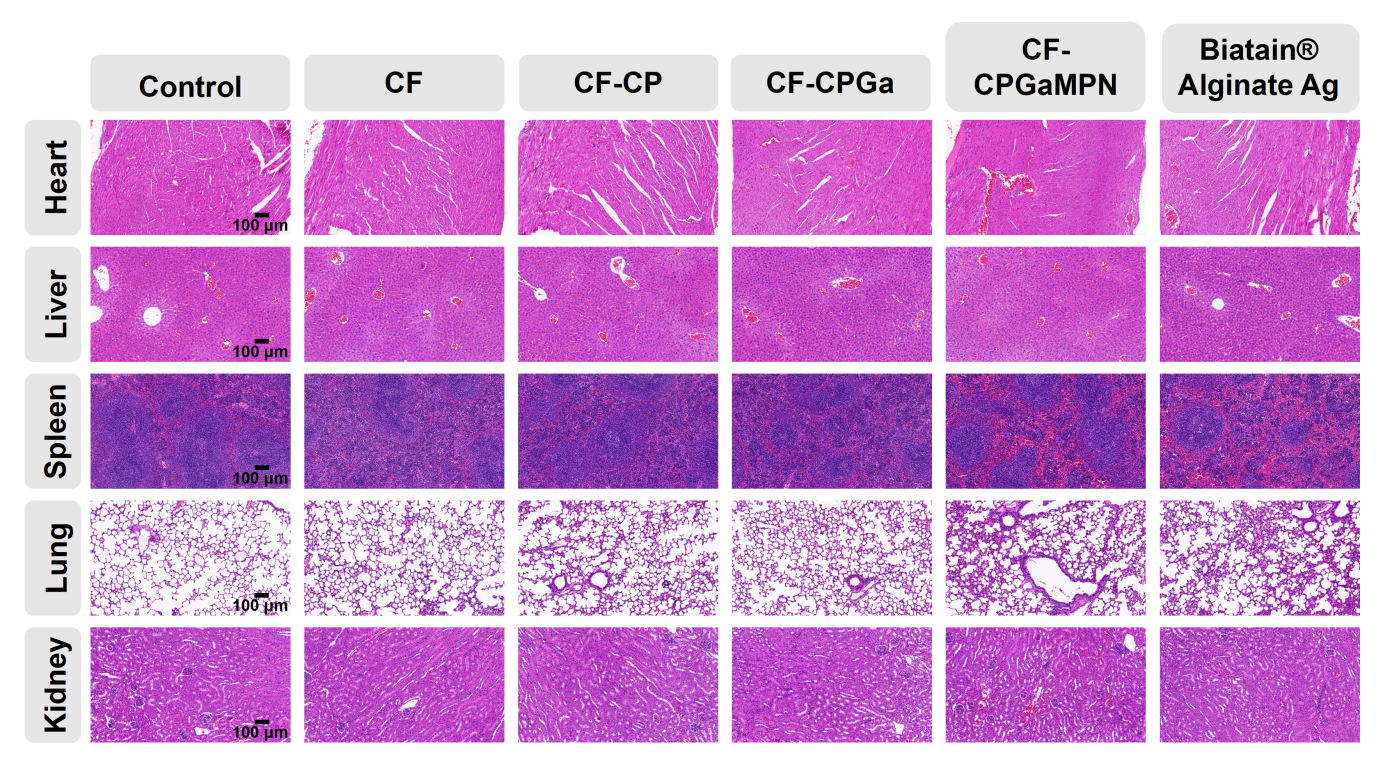


**Figure S7.** The HE staining images of the main organs of the mice after four weeks of treatment with each group of hydrogels.


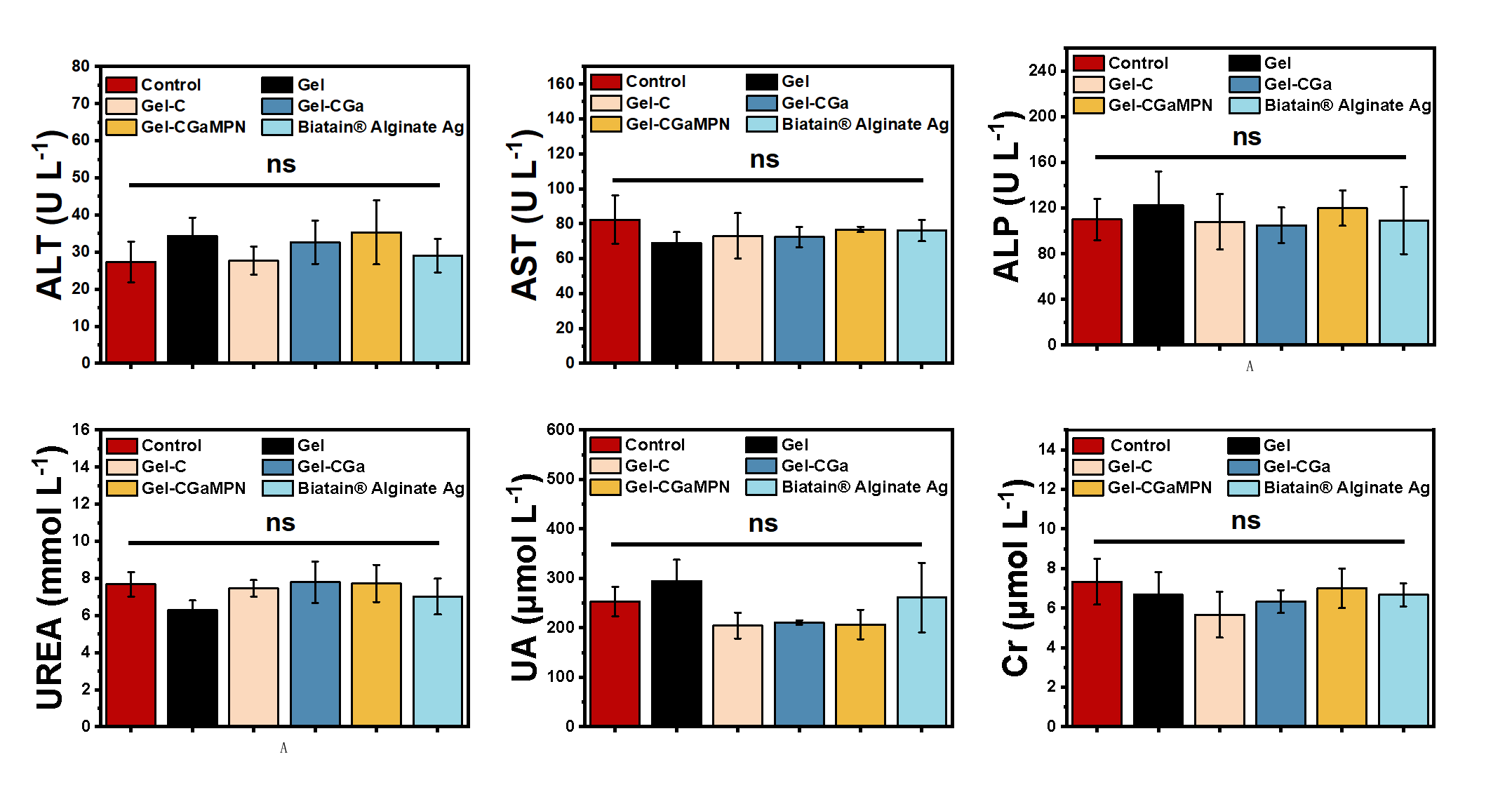


**Figure S8.** The blood biochemical indicators of the mice after four weeks of treatment with each group of hydrogels


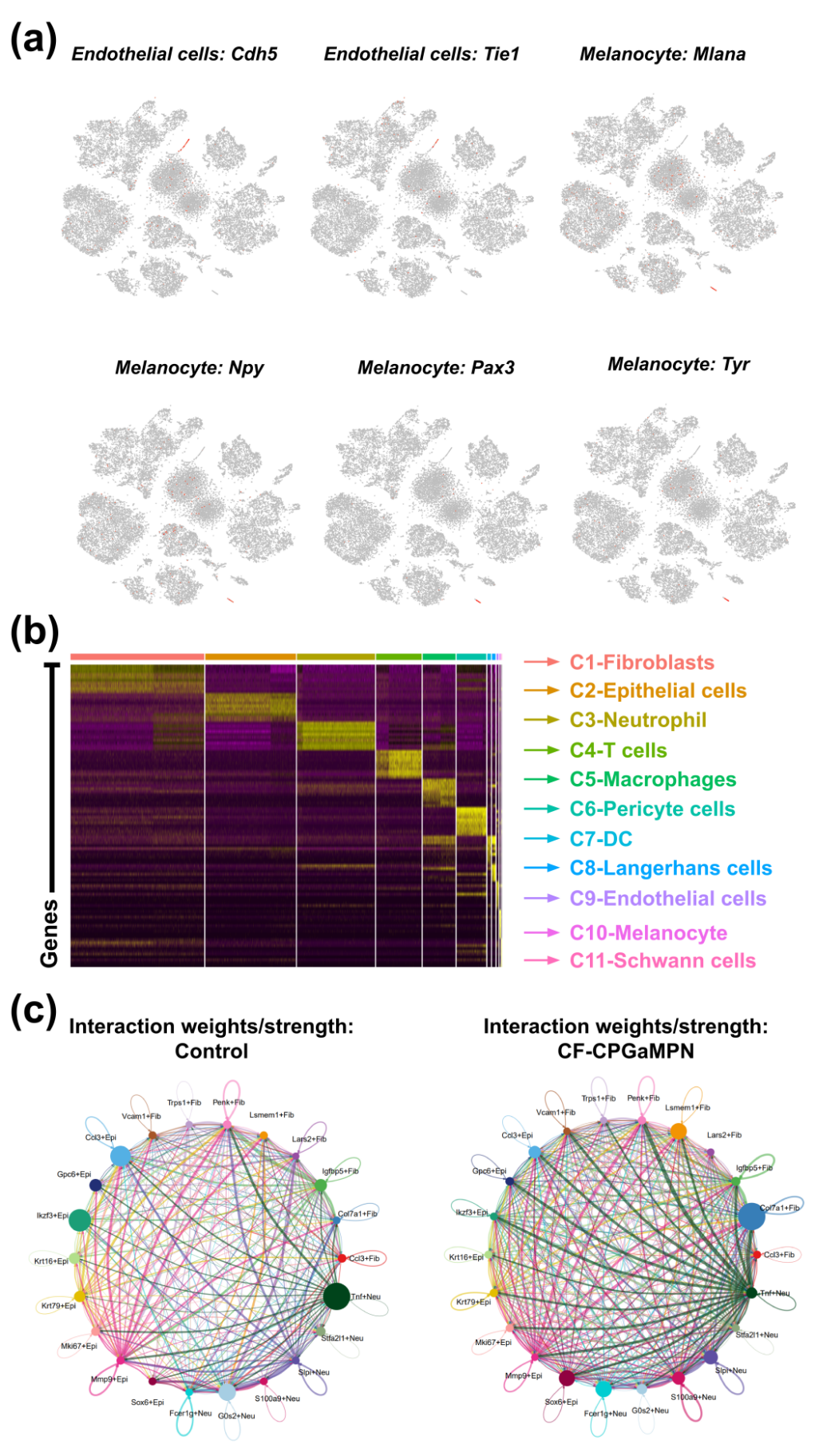


**Figure S9.** scRNA-seq analysis of wound cells on day 7. (a) Expression of selected genes across diabetic wound cell populations. (b) Unsupervised hierarchical clustering heatmap of all diabetic wound cell clusters based on the average expression of highly variable genes (correlation distance metric, average linkage). (c) CellChat was used to compare the overall cell-to-cell interaction strengths of Neutrophils, Fibroblasts and Epidermal cells in the Control and CF-CPGaMPN hydrogel groups.


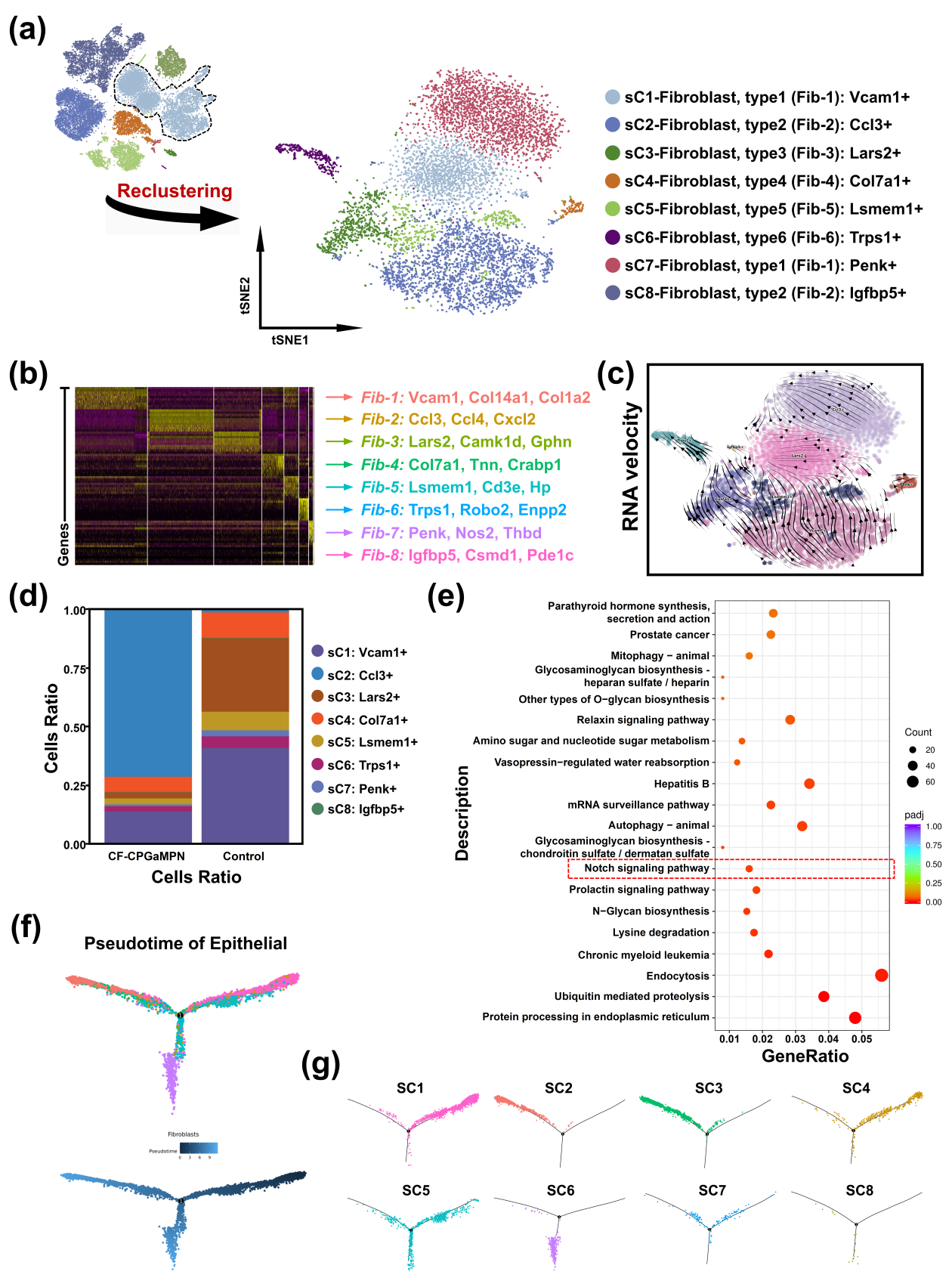


**Figure S10.** Identification of Fibroblast subclusters in hydrogels-regulated microenvironment. (a) The t-SNE plot showed re-clustering of Fibroblasts with six distinct subclusters of cells identified and color-coded. General identity of each subcluster was defined on the right. (b) Heatmap of differentially expressed genes for Fibroblasts. Selected genes for each cluster are color-coded and shown on the right. (c) RNA velocity of Fibroblasts estimated from unspliced and spliced transcripts of nearby cells and visualized on the t-SNE projection; color coded by clusters. (d) Histograms of the proportion of Fibroblast subclusters in the control and CF-CPGaMPN hydrogel groups. (e) KEGG enrichment analysis of descending genes in CF-CPGaMPN hydrogelsets. (f,g) Pseudotime lineage trajectory analysis demonstrating the relationships of subclusters among Fibroblasts (f), color coded by subclusters (g).


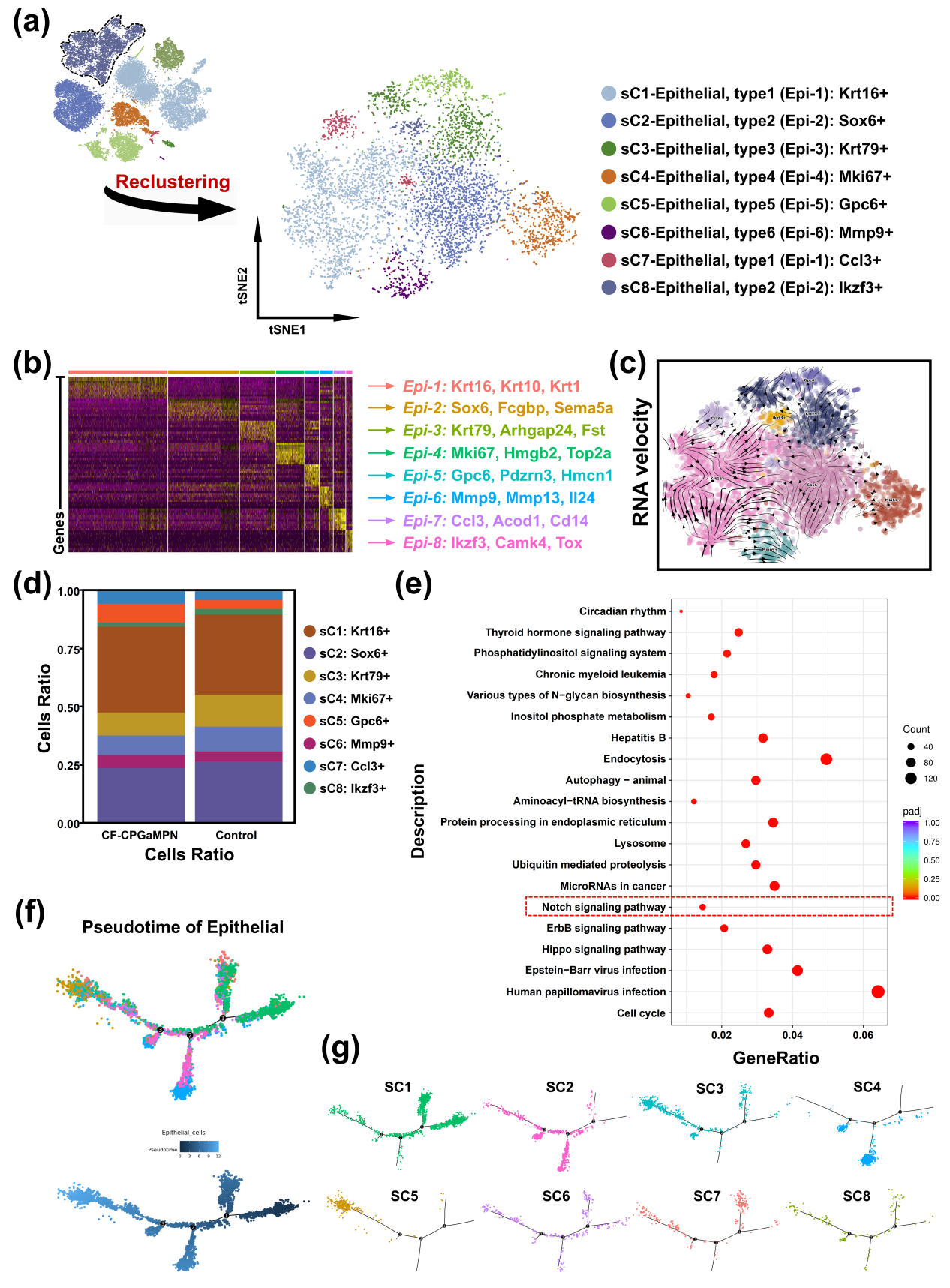


**Figure S11.** Identification of Epithelial subclusters in hydrogels-regulated microenvironment. (a) The t-SNE plot showed re-clustering of Epithelials with six distinct subclusters of cells identified and color-coded. General identity of each subcluster was defined on the right. (b) Heatmap of differentially expressed genes for Epithelials. Selected genes for each cluster are color-coded and shown on the right. (c) RNA velocity of Epithelials estimated from unspliced and spliced transcripts of nearby cells and visualized on the t-SNE projection; color coded by clusters. (d) Histograms of the proportion of Epithelial subclusters in the control and CF-CPGaMPN hydrogel groups. (e) KEGG enrichment analysis of descending genes in CF-CPGaMPN hydrogelsets. (f,g) Pseudotime lineage trajectory analysis demonstrating the relationships of subclusters among Epithelials (f), color coded by subclusters (g).


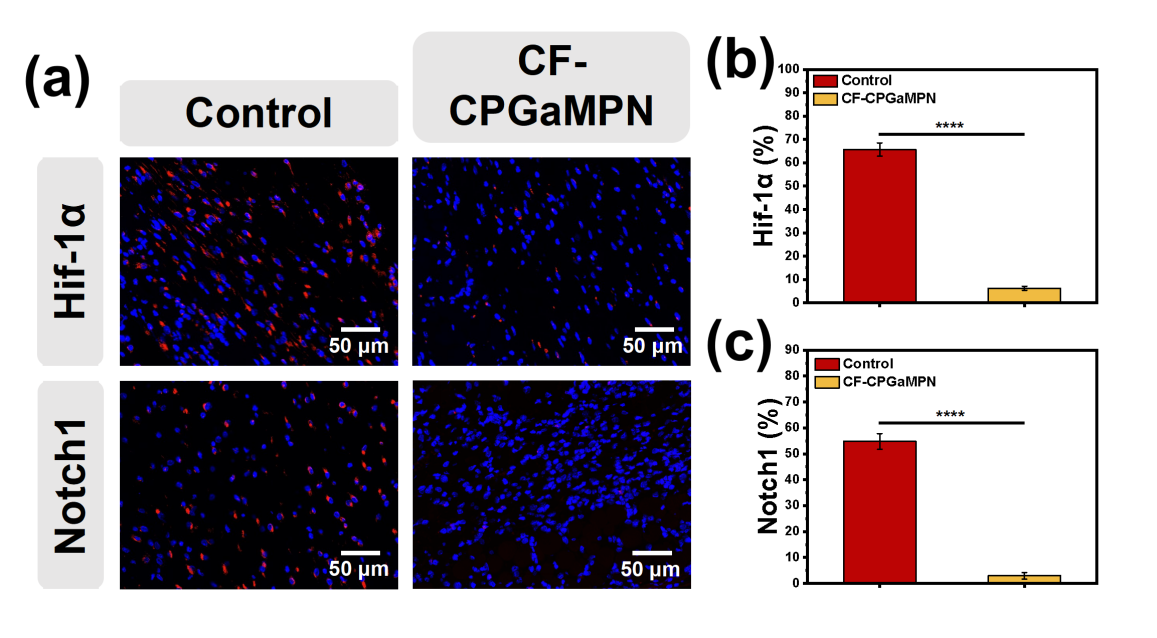


**Figure S12.** The fluorescence staining results of the infected skin tissue. (a) Immunofluorescence images of Hif-1α and Notch1 expressed in wound tissue sections. (b) Quantitative data of the percentage of Hif-1α. (c) Quantitative data of the percentage of Notch1. *p < 0.05, **p < 0.01, ***p < 0.001, ****p < 0.0001, and n = 3.
